# Rapid Characterization and Identification of Chemical Constituents in *Gentiana radix* before and after Wine-Processed by UHPLC-LTQ-Orbitrap MS^n^

**DOI:** 10.3390/molecules23123222

**Published:** 2018-12-06

**Authors:** Xin Lv, Jian-Zhi Sun, Shi-Zhao Xu, Qian Cai, Yu-Qiang Liu

**Affiliations:** Department of Medicine, Liaoning University of Traditional Chinese Medicine, Dalian 116600, China; lvxiaoxin1222@163.com (X.L.); 15804268722@163.com (J.-Z.S.); dazhao666@163.com (S.-Z.X.); liuyuqiang@126.com (Y.-Q.L.)

**Keywords:** UHPLC-LTQ-Orbitrap, iridoids, flavonoids, xanthones, characteristic fragmentation pathways, wine-processing, *Gentiana radix*

## Abstract

*Gentiana radix* is used in traditional Chinese medicine and has functions of clearing heat and drying dampness, as well as purging liver and gallbladder fire. A highly sensitive and effective strategy for rapid screening and identification of target constituents has been developed by using ultra high-performance liquid chromatography coupled with linear ion trap-Orbitrap mass spectrometry (UHPLC-LTQ-Orbitrap) in crude and wine-processed *Gentiana radix*. Based on the accurate mass measurement (<5 ppm), retention times, and MS fragmentation ions, 52 constituents were unambiguously or tentatively characterized from *Gentiana radix*, including 21 iridoids, 11 flavonoids, 19 xanthones, and a triterpenoid. This study demonstrated that the established method could be a rapid, effective analytical tool for screening and characterization of compounds in the complex systems of *Gentiana radix*. By comparing the structure and peak areas of chemical constituents in crude and wine-processed *Gentiana radix*, we found that some compounds in crude and wine-processed *Gentiana radix* were significantly different.

## 1. Introduction

*Gentiana radix* is the dried root and rhizome of *Gentiana manshurica* Kitag., *Gentiana radix* Bge., *Gentiana triflora* Pall. and *Gentiana rigescens* Franch. [1]. *Gentiana radix* is mainly distributed in Heilongjiang, Jilin, Liaoning, Nei Monggol, Zhejiang, Jiangsu, Guangdong, Yunnan, Xinjiang, etc. [2]. *Gentiana radix* is commonly employed to treat various diseases. For instance, *Gentiana radix* could be used for treating heat jaundice, pruritus and swells of vulvaered, morbid leucorrhea, eczema, tinnitus, epicophosis, hypochondriac pain, invigorating stomach and convulsions. Modern pharmacological studies have demonstrated that *Gentiana radix* possesses various biological activities, including anti-inflammatory, anti-oxidative and antiviral [3,4]. *Gentiana radix* has complicated chemical constituents, including iridoid glycoside, flavonoid glycoside, xanthones, triterpenoids, alkaloids, and so on. Among them, iridoid glycoside, flavonoid glycoside and xanthones are considered the main constituents [5]. There are many processing methods involving *Gentiana radix* recorded in history, such as wine-processing, bile-processing, honey-processing and ginger-processing [6,7,8]. And now crude and wine-processed *Gentiana radix* is widely used in clinical practice. According to traditional theory, wine-processing could make the function of the drug move upward, and wine-processing alleviated the bitterness and coldness of crude *Gentiana radix*. Physical and chemical changes have taken place in the medicinal materials after being wine-processed so that it would lead to changes in the content and types of chemical constituents [9].

In recent years, high-resolution mass spectrometry (HRMS) and linear ion trap-Orbitrap mass spectrometer (LTQ-Orbitrap) have been exhibiting excellent (i.e. fast and sensitive) performance in traditional Chinese medicines (TCMs) extracts [10,11]. The hybrid linear ion trap-Orbitrap mass spectrometer (LTQ-Orbitrap) has high quality resolution and mass accuracy (within 5 ppm), it combines the high trapping capability with MS^n^ and scanning capability of a linear ion trap [12,13]. LTQ-Orbitrap allows the effective detection of a large amount of data about chemical constituents, and it includes exact mass, elemental compositions, fragmentation pathways, etc. [14]. These capabilities play a vital role in effectively identifying and analyzing the complicated constituents of TCMs. In this paper, a method with UHPLC-LTQ-Orbitrap was established to comprehensively analyze the constituents in *Gentiana radix*, and this method is applied to the comparative study of the constituents in crude and wine-processed *Gentiana radix*. Fifty-two constituents were identified, the similarities and differences of the constituents in *Gentiana* before and after wine processing were determined.

## 2. Results and Discussion

### 2.1. Identification of the Constituents by UHPLC-LTQ-Orbitrap MS^n^

**Figure 1:**
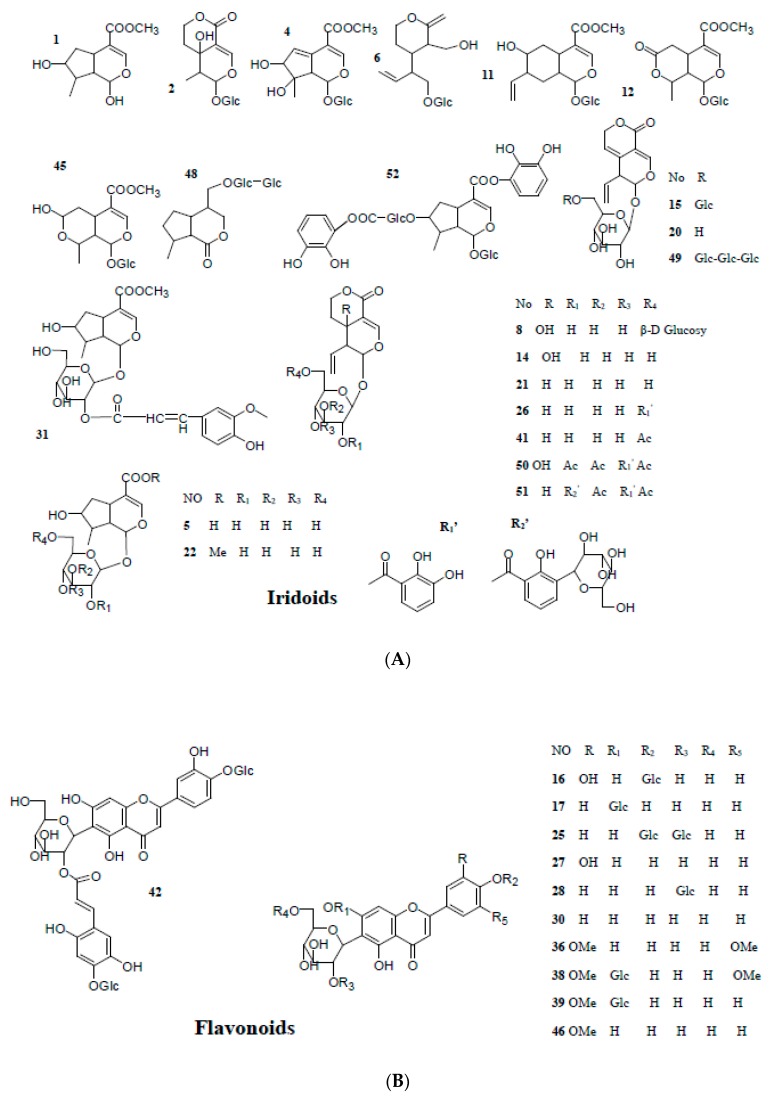

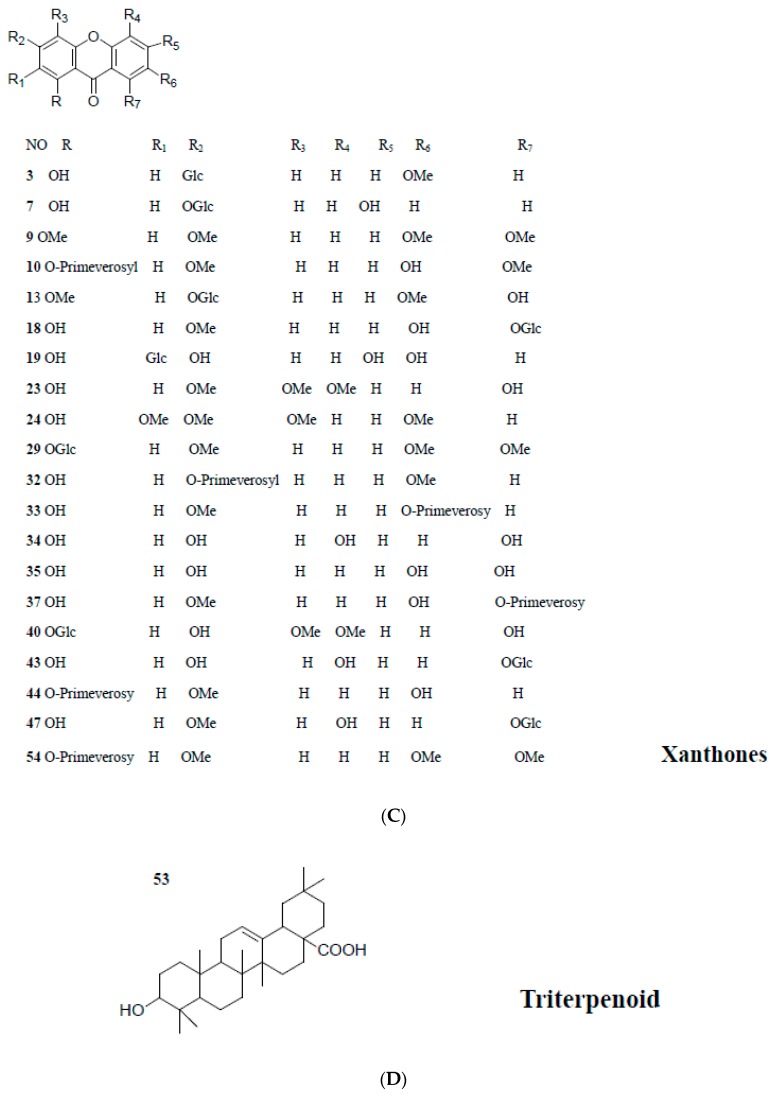
The structures of compounds identified in crude and wine-processed *Gentiana radix*: (**A**) iridoids; (**B**) flavonoids; (**C**) xanthones; (**D**) triterpenoid.

**Figure 2:**
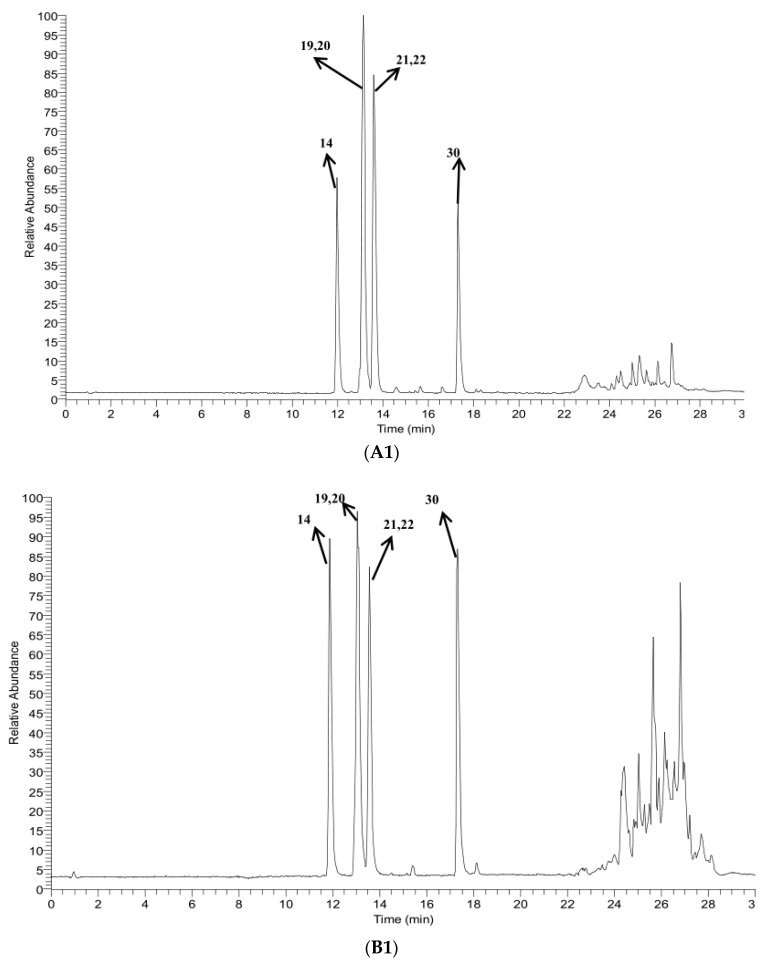

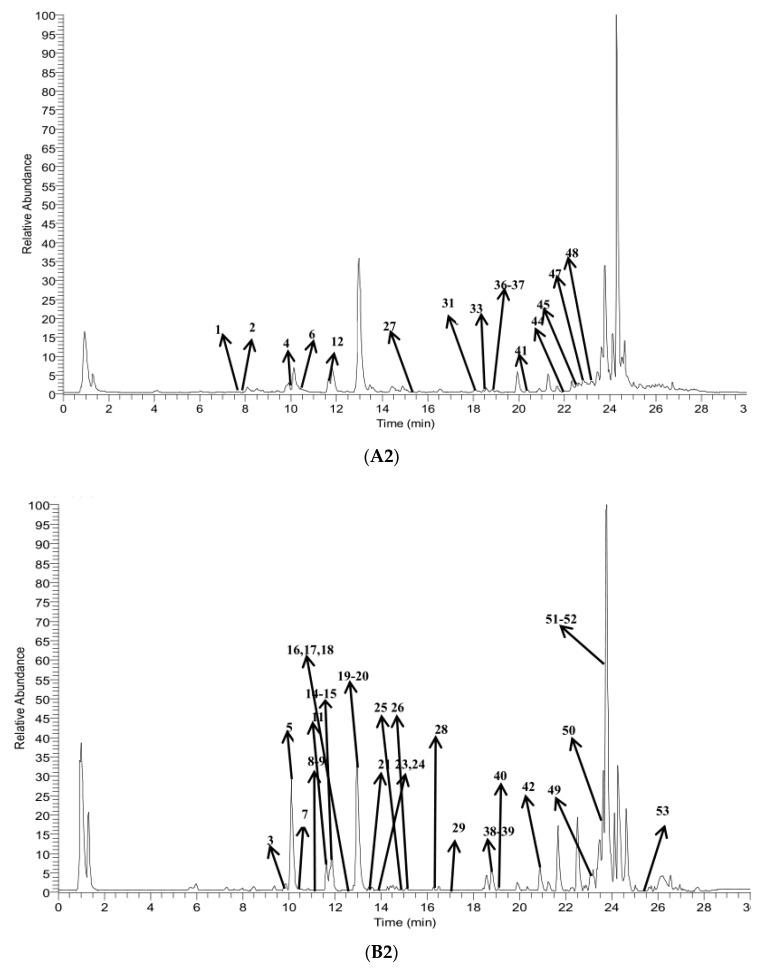

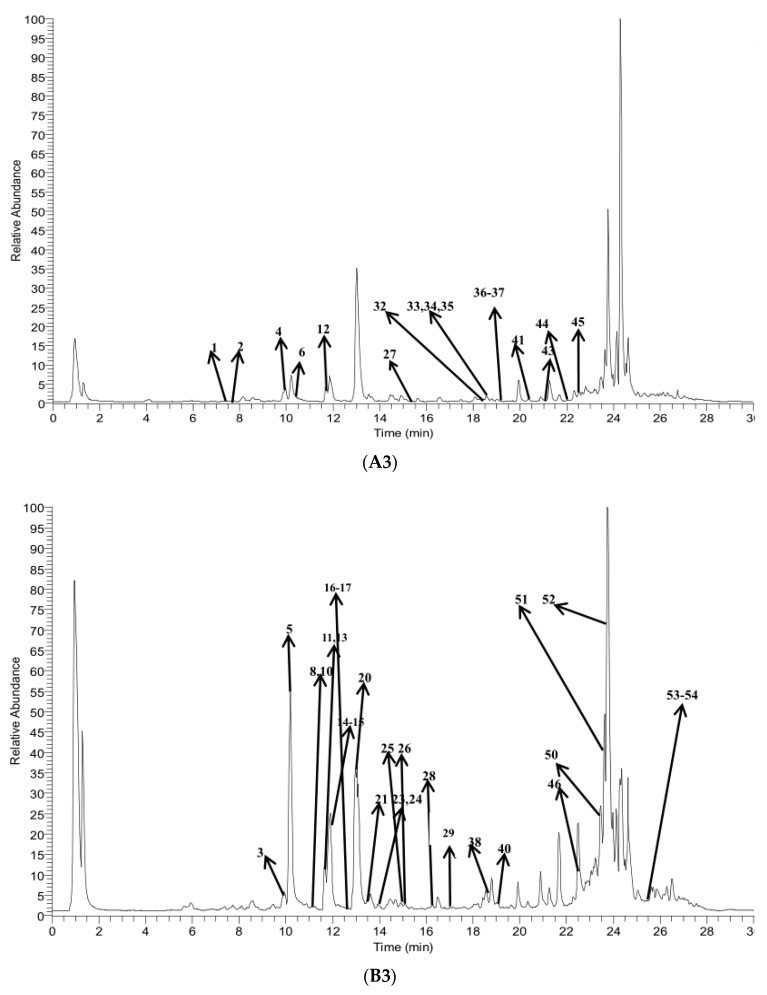
TIC chromatogram of standards, crude and wine-processed *Gentiana radix:* (**A**) positive ion mode; (**B**) negative ion mode; (**1**) standards; (**2**) crude *Gentiana radix*; (**3**) wine-processed *Gentiana radix.*

UHPLC-ESI-LTQ-Orbitrap was employed for comprehensive analysis in positive and negative modes to identify the chemical constituents in *Gentiana radix*. In order to determine the molecular formula of compounds, it was necessary to compare it with the HRMS molecular formula database built in-home, the high-accuracy protonated precursors with errors less than 5 ppm and related literature. As a result, a total of 52 compounds (Table 1, Figure 1) were screened and identified from *Gentiana radix* extract, including 21 iridoids, 11 flavonoids, 19 xanthones and a triterpenoid. By comparing the constituents in crude and wine-processed *Gentiana radix,* the results showed that there were 34 identical chemical constituents in them, 10 constituents characteristic of crude *Gentiana radix*, and 8 constituents characteristic of wine-processed *Gentiana radix.* And the peak area of some constituents increased or decreased after *Gentiana radix* was processed with wine. A typical total ion chromatogram (TIC) of crude and wine-processed *Gentiana radix* in positive and negative ion mode is showed in Figure 2.

#### 2.1.1. Structural Characterization and Identification of Iridoid

Compound 1 produced the [M + H]^+^ ion at *m*/*z* 229.10652 and 229.10654 (C_11_H_17_O_5_, mass error = 2.314 ppm and 2.227 ppm). In the MS^2^ spectrum, they all produced ions at *m*/*z* 211 [M + H − H_2_O]^+^, *m*/*z* 193 [M + H − H_2_O − H_2_O]^+^, suggesting the presence of two hydroxyl groups, *m*/*z* 211 was identified as the base peak. In addition, all of them produced [M + H − CH_3_OH]^+^ and [M + H − CH_3_OH − CO]^+^ions at *m*/*z* 197 and *m*/*z* 169, suggested the presence of carbomethoxy [40]. It yielded a series of ions at *m*/*z* 179 [M + H − H_2_O − CH_3_OH]^+^, *m*/*z* 151 [M + H − H_2_O − CH_3_OH − CO]^+^, *m*/*z* 161 [M + H − H_2_O − H_2_O − CH_3_OH]^+^, *m*/*z* 133 [M + H − H_2_O − H_2_O − CH_3_OH − CO]^+^. By comparison with the reference standard, compound 1 was predicatively annotated as loganetin.

Compound 2 showed the [M + H]^+^ ion at *m*/*z* 391.12317 and 391.12280 (C_16_H_23_O_11_, mass error = 0.813 ppm and 1.759 ppm). In the MS^2^ spectrum, *m*/*z* 229 [M + H − Glc]^+^ was identified as the base peak. Other product ions like *m*/*z* 373 [M + H − H_2_O]^+^, *m*/*z* 211 [M + H − H_2_O − Glc]^+^ and *m*/*z* 193 [M + H − H_2_O – Glc − H_2_O]^+^. The ion at *m*/*z* 125 [M − 266]^+^ was yielded by RDA cleavage fragmentation at 5-position of the O-ring and 7-position of the C-ring. By comparison with the literature data, compound 2 was tentatively identified as eustomoside.

Compound 4 showed the [M + H]^+^ ion at *m*/*z* 405.13898 and 405.13864 (C_17_H_25_O_11_, mass error = 0.390 ppm and 1.229 ppm). It generated a serial of ions at *m*/*z* 387 [M + H − H_2_O]^+^, *m*/*z* 373 [M + H − CH_3_OH]^+^, *m*/*z* 345 [M + H − CH_3_OH − CO]^+^, *m*/*z* 243 [M + H − Glc]^+^, *m*/*z* 225 [M + H − Glc − H_2_O]^+^, *m*/*z* 211 [M + H − Glc − CH_3_OH]^+^, *m*/*z* 193 [M + H − Glc − CH_3_OH − H_2_O]^+^, *m*/*z* 175 [M + H − Glc − CH_3_OH − H_2_O − H_2_O]^+^ and *m*/*z* 165 [M + H − Glc − H_2_O − CH_3_OH − CO]^+^. Thus, compound 4 was tentatively determined as methyl (1*S*)-1-(d-glucopyranosyloxy)-6,7-dihydroxy-7-methyl-1,6,7,7a-tetrahydrocyclopenta[c]pyran-4-carboxylate.

**Figure 3:**
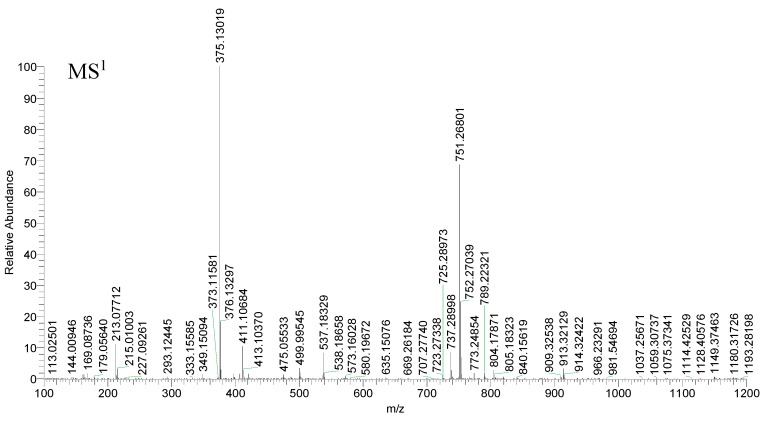

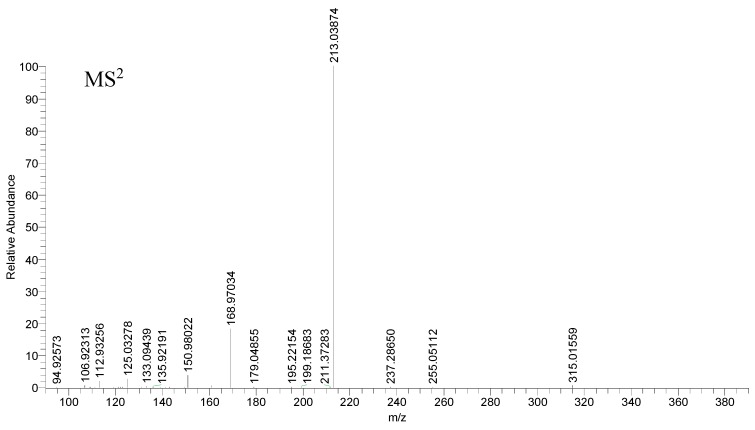
Spectra of ion fragments in MS^n^ analysis of loganic acid in negative ion mode.

**Figure 4:**
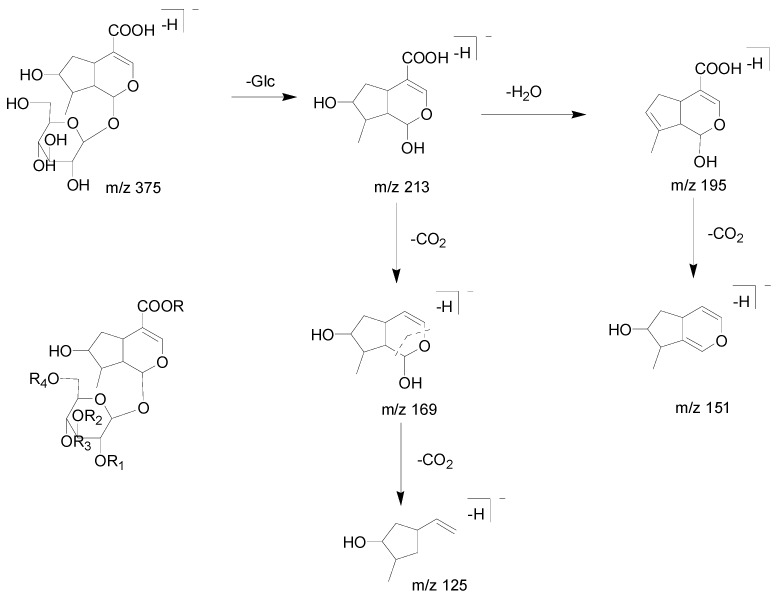
The proposed fragmentation pathway of loganic acid.

Compound 5 yielded its quasi-molecular ions [M − H]^−^ at *m*/*z* 375.13019 and *m*/*z* 375.13022 (mass error = 4.310 ppm and mass error = 4.390 ppm) (C_16_H_23_O_10_), respectively. Both of them generated the same ESI-MS^2^ ions at *m*/*z* 213 [M − H − Glc]^−^, *m*/*z* 195 [M − H − Glc − H_2_O]^−^, *m*/*z* 169 [M − H − Glc − CO_2_]^−^, 151 [M − H − Glc − H_2_O − CO_2_]^−^ and *m*/*z* 125 [M − H − Glc − 2CO_2_]^−^ [41]. The proposed spectra of chromatograms of fragmentation MS^n^ of compound 5 is shown in Figure 3, and the proposed fragmentation pathway of compound 5 is shown in Figure 4. So, compound 5 was tentatively determined as loganic acid [42].

Compound 6 showed the [M + Na]^+^ at *m*/*z* 385.14673 and *m*/*z* 385.14664 (C_16_H_26_O_9_Na, mass error = 0.450 ppm and mass error = 0.684 ppm). It generated a serial of ions at *m*/*z* 367 [M + Na − H_2_O]^+^, *m*/*z* 357 [M + Na − CO]^+^, *m*/*z* 339 [M + Na − CO − H_2_O]^+^, *m*/*z* 355 [M + Na − HCOH]^+^, *m*/*z* 223 [M + Na − Glc]^+^ and *m*/*z* 205 [M + H − Glc − H_2_O]^+^. By comparison with the reference standard, compound 6 was predicatively annotated as 1-*O*-d-Glucopyransylampexine.

**Figure 5:**
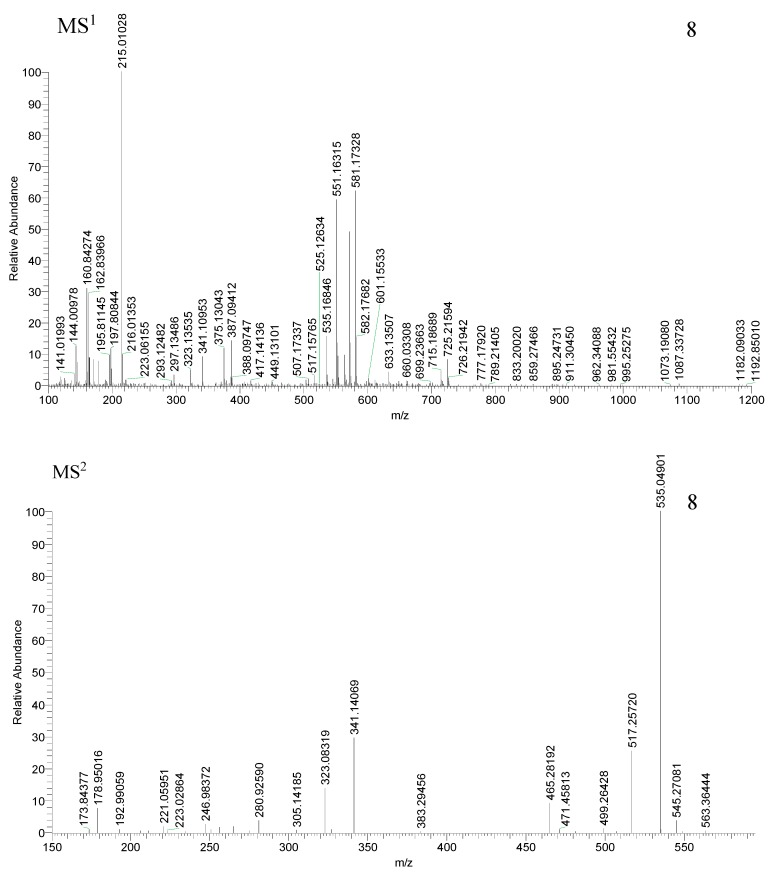

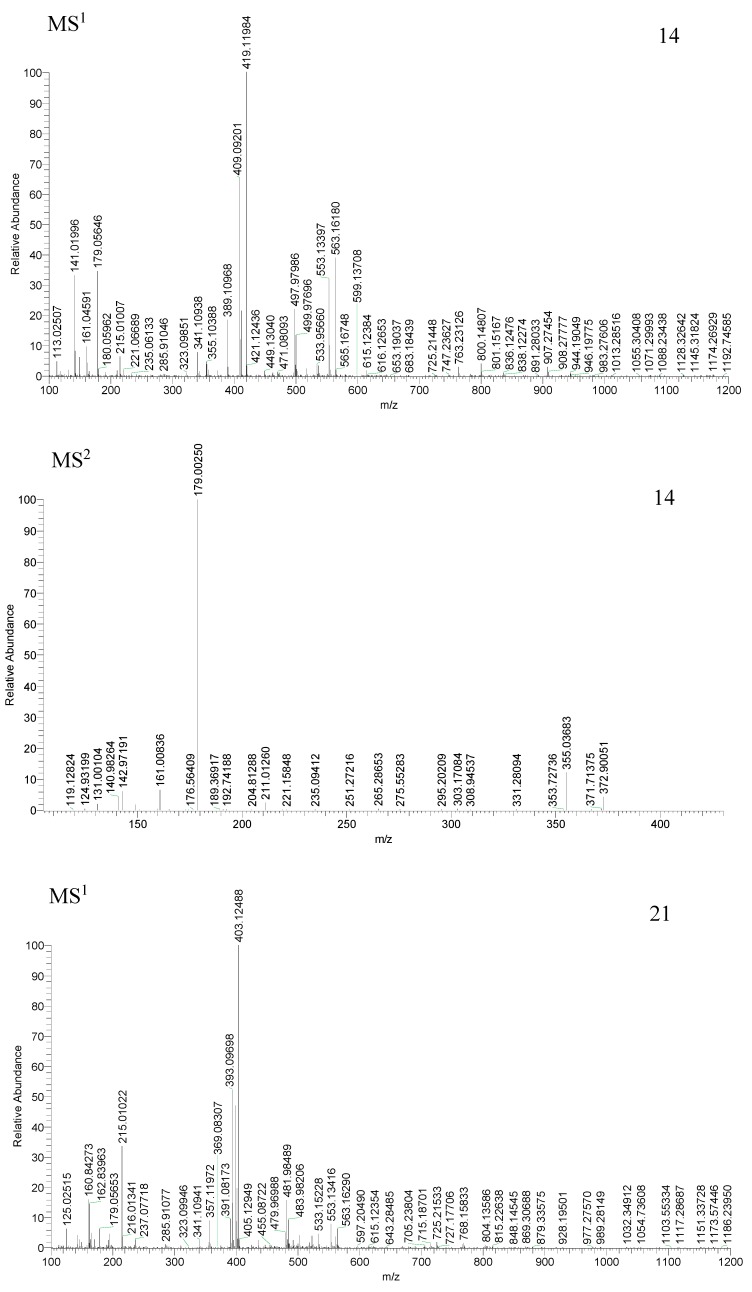

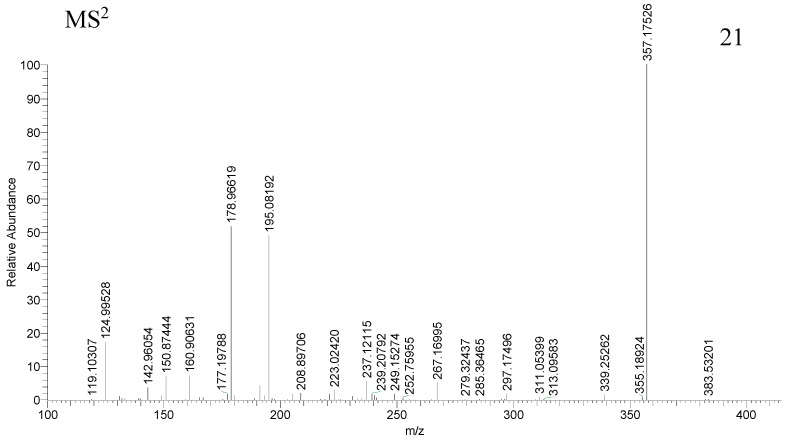
Spectra of ion fragments in MS^n^ analysis of 6′-*O*-β-d-glucosyl swertiamarin (**8**), swertiamarin (**14**), sweroside (**21**) in negative ion mode.

**Figure 6:**
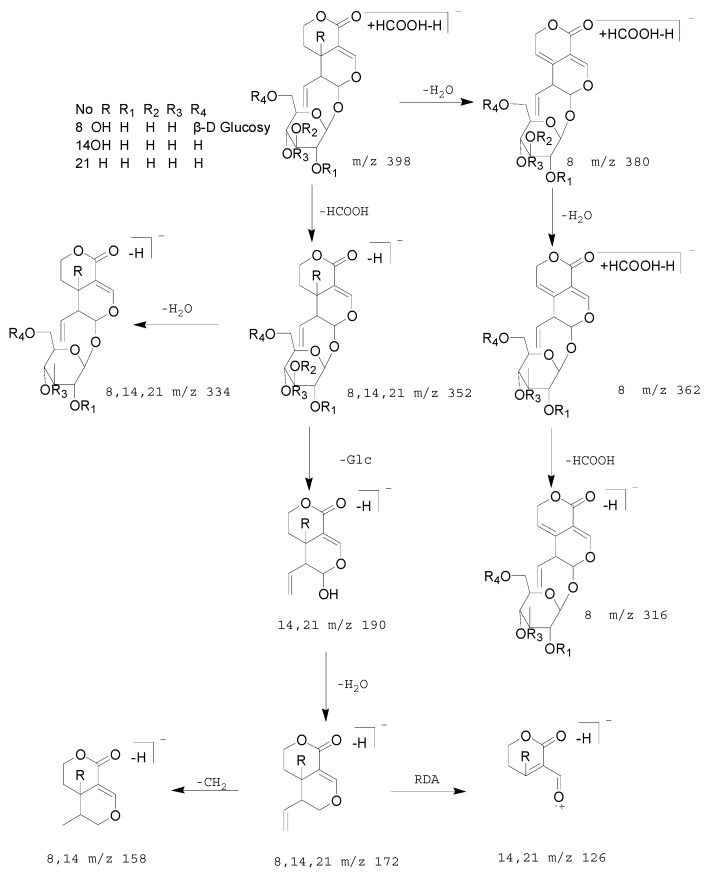
The proposed fragmentation pathway of 6′-*O*-β-d-glucosyl swertiamarin, swertiamarin, sweroside.

Compounds 8, 14, and 21 produced their [M − H + HCOOH]^−^ ions at *m*/*z* 581.17352 and 581.17328 (C_23_H_33_O_17_, mass error = 3.948 ppm and mass error = 3.535 ppm), 419.11990 and 419.11984 (C_17_H_23_O_12_, mass error = 3.573 ppm and mass error = 3.430 ppm), and 403.12506 and 403.12488 (C_17_H_23_O_11_, mass error = 1.5723 ppm and mass error=1.392 ppm), respectively. All of them generated [M − H]^−^ and [M − H − H_2_O]^−^ ion at *m*/*z* 535, *m*/*z* 373, *m*/*z* 357, *m*/*z* 517, *m*/*z* 355, *m*/*z* 339, respectively. Compounds 8 and 14 also generated the [M − H − Glc − H_2_O − CH_2_]^−^ ion at *m*/*z* 341, *m*/*z* 179. Compounds 14 and 21 also generated the [M − H − Glc]^−^, [M − H − Glc − H_2_O]^−^ and [M − H − neutral molecule]^−^ (RDA) ion at *m*/*z* 211, *m*/*z* 195, *m*/*z* 193, *m*/*z* 177, *m*/*z* 151, *m*/*z* 141, *m*/*z* 125. The MS^2^ ions at *m*/*z* 193 (C_10_H_11_O_5_) were generated by cleavage fragmentation from 1-position of quasi-molecular ions in compounds 8 and 14. Compound 8 generated [M − H + HCOOH − H_2_O]^−^, [M − H + HCOOH − H_2_O − H_2_O]^−^ and [M − H − H_2_O − H_2_O]^−^ ions at *m*/*z* 563, *m*/*z* 545 and *m*/*z* 499. The fragmentation pathways were consistent with deduced of compounds 8, 14 and 21, which further proved the validity of the results (Figure 5 and Figure 6). Therefore, compound 8 was tentatively deduced as 6′-*O*-β-d-glucosyl swertiamarin, compared with the t_R_ values and mass spectra with the reference standard, 14 and 21 were tentatively annotated as swertiamarin and sweroside [43].

Compound 11 generated [M − H]^−^ ion at *m*/*z* 389.14285 and 389.14258 (C_17_H_25_O_10_, mass error = 3.529 ppm and mass error = 4.223 ppm).The [M − H]^−^ ion produced the ions at *m*/*z* 371, *m*/*z* 345 and *m*/*z* 209 in the MS^2^ spectrum, which originated from the neutral loss of H_2_O, CO_2_ and a glucose. In addition, the molecular ion also produced the minor ion at *m*/*z* 191 [M − H − Glc − H_2_O − H_2_O]^−^ and *m*/*z* 163 [M − H − Glc − H_2_O − H_2_O − CO]^−^. Thus, compound 11 was tentatively determined as secologanol.

Compounds 12 and 45 produced the [M + H]^+^ ions at *m*/*z* 405.13895 and *m*/*z* 405.13858 (C_17_H_25_O_11_, mass error = 0.188 ppm and mass error = 0.558 ppm), *m*/*z* 407.15421 and *m*/*z* 407.15466 (C_17_H_27_O_11_, mass error = 1.125 ppm and mass error = 0.314 ppm). Its MS^2^ spectrum produced ions at *m*/*z* 387, *m*/*z* 369, *m*/*z* 389 and *m*/*z* 371, which involved the loss of one and two molecules of H_2_O, respectively. In addition, compounds 12 and 45 also produced a serial of ions at *m*/*z* 243 and 245 [M + H − Glc]^+^, *m*/*z* 225 and 227 [M + H − Glc − H_2_O]^+^, *m*/*z* 193 and 195 [M + H − Glc − H_2_O − CH_3_OH]^+^, *m*/*z* 183 and 185 [M + H − Glc − CH_3_OH − CO]^+^, *m*/*z* 165 and 167 [M + H − Glc − H_2_O − CH_3_OH − CO]^+^ [44]. Compound 50 produced the [M + H − Glc − CH_3_OH]^+^ ions at *m*/*z* 211. So, compounds 12 and 45 were tentatively determined as kingiside and morroniside.

Compounds 15 and 20 generated their [M − H + HCOOH]^−^ ions at *m*/*z* 563.16193 and *m*/*z* 563.16144 (C_23_H_31_O_16_, mass error = 2.168 ppm and mass error = 2.048 ppm), *m*/*z* 401.10907and *m*/*z* 401.10892 (C_17_H_21_O_11_, mass error=1.232 ppm and mass error = 1.082), respectively. Both of their deprotonated molecular ions produced [M − H]^−^, [M − H − Glc]^−^, [M − H − Glc − CO]^−^, [M − H − Glc − CO − H_2_O]^−^ and [Glu − H]^−^ ions at *m*/*z* 517, *m*/*z* 355, *m*/*z* 355, *m*/*z* 193, *m*/*z* 327, *m*/*z* 165, *m*/*z* 309, *m*/*z* 149, *m*/*z* 341, and *m*/*z* 179, respectively [45]. In addition, compound 20 could generate the ion at *m*/*z* 175 by losing of H_2_O and a glucose. However, compound 15 produced ions at *m*/*z* 193 [M − H − Glc − Glc]^−^, *m*/*z* 165 [M − H − Glc − Glc − CO]^−^, *m*/*z* 323 [M − H − Glc − H_2_O − CH_2_]^−^ and *m*/*z* 305 [M − H − Glc − H_2_O − CH_2_ − H_2_O]^−^. So, compound 15 was tentatively ascertained as 6′-*O*-β-d-glucosyl gentiopicroside, compared with the t_R_ values and mass spectra with the reference standard, compound 20 was tentatively ascertained as gentiopicroside.

Compound 22 generated their [M − H + HCOOH]^−^ ion at *m*/*z* 435.15146 (C_18_H_27_O_12_, mass error = 4.039 ppm), respectively. Compound 22 could generate ions at *m*/*z* 389 [M − H]^−^, *m*/*z* 371 [M − H − H_2_O]^−^, *m*/*z* 227 [M − H − Glc]^−^, *m*/*z* 209 [M − H − Glc − H_2_O]^−^ and *m*/*z* 127 (C_7_H_11_O_2_, RDA ). So, compound 22 was tentatively ascertained as loganin.

Compounds 26, 50, and 51 generated their [M − H]^−^ ions at *m*/*z* 655.18951 and 655.18927 (C_29_H_35_O_17_, mass error = 4.020 ppm and mass error = 3.654 ppm), *m*/*z* 797.21667 and 797.21637 (C_35_H_41_O_21_, mass error = 3.996 ppm and mass error = 3.620 ), *m*/*z* 875.22638 and 875.22614 (C_40_H_43_O_22_, mass error = 2.663 ppm and mass error = 3.413ppm), respectively. The ions at *m*/*z* 315 (C_15_H_19_O_10_) were generated by α-cleavage fragmentation of [M − H]^−^ ion and loss a neutral molecule (C_2_H_2_O, *m*/*z* 42). Both of their deprotonated molecular ions produced [M − H − H_2_O]^−^ and [M − H − Glc]^−^ ions at *m*/*z* 637, *m*/*z* 779, *m*/*z* 857, *m*/*z* 493, *m*/*z* 635, *m*/*z* 713. Compounds 26 and 51 also generated the [M − H − R_1_′]^−^, ions at *m*/*z* 577, *m*/*z* 357. And compounds 50 and 51 generated the [M – H − CH_3_OH − CO]^−^ and [M − H − CH_3_OH − CO − Glc]^−^ ions at *m*/*z* 815, *m*/*z* 737, *m*/*z* 653, *m*/*z* 575. Compounds 26 and 50 generated the [M − H− Glc − H_2_O]^−^ ions at *m*/*z* 475, *m*/*z* 617 [46]. In addition, compound 26 also produced the minor ion at *m*/*z* 195 [M − H − R_1_′ − Glc]^−^. Compound 50 produced [M − H − 2H_2_O]^−^, [M − H − CH_3_OH]^−^, [M − H − 2CH_3_OH − 2CO]^−^, [M − H − 3CH_3_OH − 3CO]^−^, [M − H − CH_3_OH − Glc]^−^, [M − H − 2CH_3_OH − 2CO − Glc]^−^ and [M − H − 3CH_3_OH − 3CO − Glc]^−^ ions at *m*/*z* 761, *m*/*z* 755, *m*/*z* 677, *m*/*z* 617, *m*/*z* 593, *m*/*z* 617, *m*/*z* 475, *m*/*z* 515, *m*/*z* 455. Compound 51 produced [M − H − R_2_′]^−^, [M − H − R_2_′− CH_3_OH − CO]^−^ [M − H − R_2_′ − CH_3_OH − CO − Glc]^−^ and [M − H − R_2_′ − R_1_′]^−^ ions at *m*/*z* 739, *m*/*z* 679, *m*/*z* 517, *m*/*z* 441. Compounds 26, 50 and 51 were predicatively annotated as gentiotrifloroside, rindoside, macrophylloside A.

Compound 31 produced its [M + H]^+^ ion at *m*/*z* 567.20477 (C_27_H_35_O_13_, mass error = 2.447 ppm). It generated a series of ions at *m*/*z* 549 [M + H − H_2_O]^+^, *m*/*z* 471 [M + H − H_2_O − CH_3_OH − CO]^+^, *m*/*z* 453 [M + H − 2H_2_O − CH_3_OH−CO]^+^, *m*/*z* 453 [M + H − 3H_2_O − CH_3_OH − CO]^+^, *m*/*z* 229 [M + H – Glc − C_10_H_8_O_3_]^+^ and *m*/*z* 211 [M + H − Glc − C_10_H_8_O_3_ − H_2_O]^+^. Therefore, it was tentatively identified as 2′-feruloy loganin.

Compound 41 generated its [M + H]^+^ ion at *m*/*z* 401.14362 and *m*/*z* 401.14392(C_18_H_25_O_11_, mass error = 1.504 ppm and mass error = 1.504 ppm). It generated a serial of ions at *m*/*z* 383 [M + H − H_2_O]^+^, *m*/*z* 365 [M + H − 2H_2_O]^+^, *m*/*z* 341 [M + H − CH_3_OH − CO]^+^, *m*/*z* 197 [M + H − C_8_H_12_O_6_]^+^, *m*/*z* 179 [M + H − C_8_H_12_O_6_ − H_2_O]^+^, *m*/*z* 151 [M + H − C_8_H_12_O_6_ − H_2_O − CO]^+^and *m*/*z* 127 (C_6_H_6_O_3_, RDA). Therefore, compound 41 was tentatively annotated as 3′-acetyl swerside.

Compound 48 generated [M + Na]^+^ ion at *m*/*z* 579.26117 (C_24_H_44_O_14_Na, mass error = 1.997 ppm). The [M + Na]^+^ ion produced the ions at *m*/*z* 561 and *m*/*z* 543 in the MS^2^ spectrum, which involved the loss of one and two molecules of H_2_O. In addition, it produced *m*/*z* 417 [M + Na − Glc]^+^, *m*/*z* 399 [M + Na − Glc − H_2_O]^+^, *m*/*z* 255 [M + Na − 2Glc]^+^, *m*/*z* 237 [M + Na − 2Glc − H_2_O]^+^ and *m*/*z* 383 [M + Na − Glc − CO − CH_4_]^+^. Thus, compound 48 was tentatively determined as gentianaside.

Compound 49 produced the [M − H]^−^ ion at *m*/*z* 841.26135(mass error = 1.356 ppm) (C_34_H_49_O_24_ ). The molecular ions yield [M − H]^−^, [M – H − Glc]^−^, [M − H − 2Glc]^−^, [M − H − 2Glc − C_2_H_4_]^−^, [M − H − 2Glc − C_2_H_4_ − 4H_2_O]^−^ and [M − H − 3Glc − C_2_H_4_]^−^, *m*/*z* 841, *m*/*z* 679, *m*/*z* 517, *m*/*z* 489, *m*/*z* 477 and *m*/*z* 337. According to the literature data, compound 49 was tentatively annotated as scabran G4.

Compound 52 generated [M − H]^−^ ion at *m*/*z* 781.22137 and *m*/*z* 781.22119 (C_35_H_41_O_20_, mass error = 3.578 ppm and 3.365 ppm). The ions at *m*/*z* 619 and *m*/*z* 601 were yielded by neutral loss one molecule of glucosyl and glucose, respectively. Moreover, the molecular ion generated ions at *m*/*z* 655 [M − H − C_6_H_6_O_3_]^−^, *m*/*z* 493 [M − H − C_6_H_6_O_3_ − Glc]^−^ and *m*/*z* 339 [M − H − C_6_H_6_O_3_ − 2Glc − H_2_O]^−^. According to the literature data, compound 52 was ascertained as depressoside.

#### 2.1.2. Structural Characterization and Identification of Flavonoid

**Figure 7:**
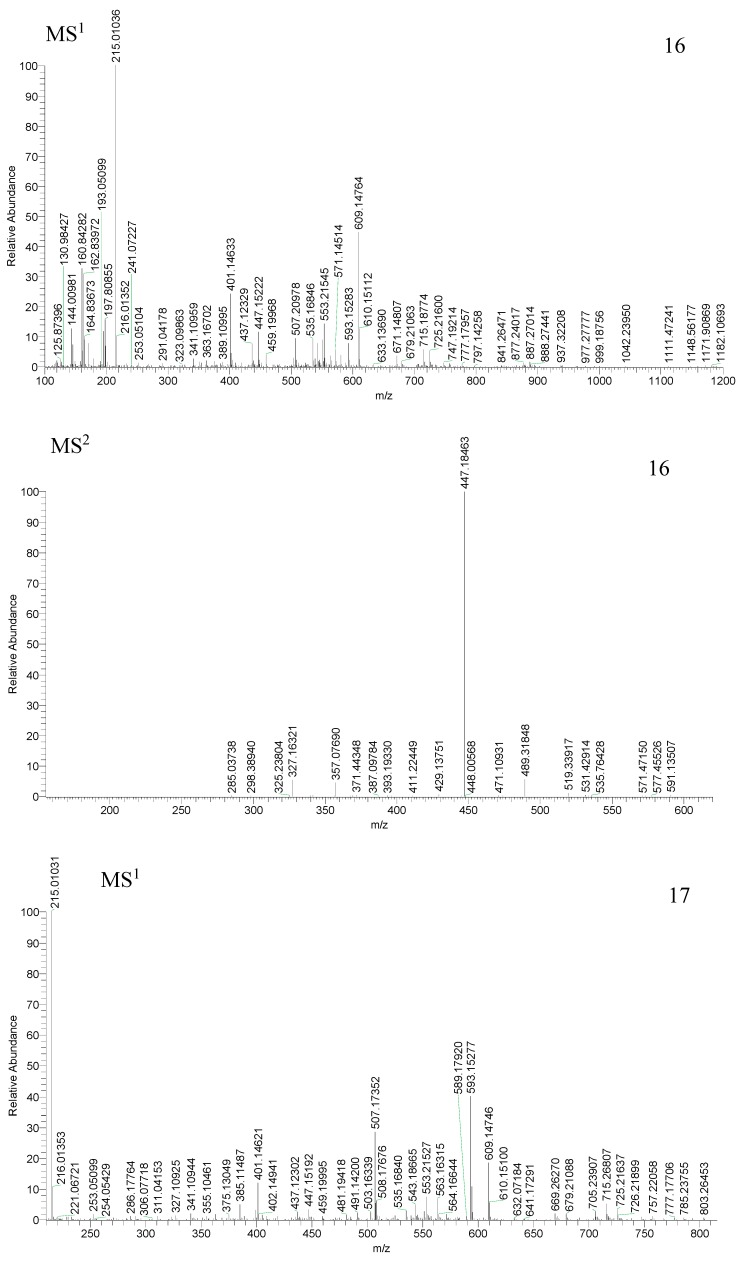

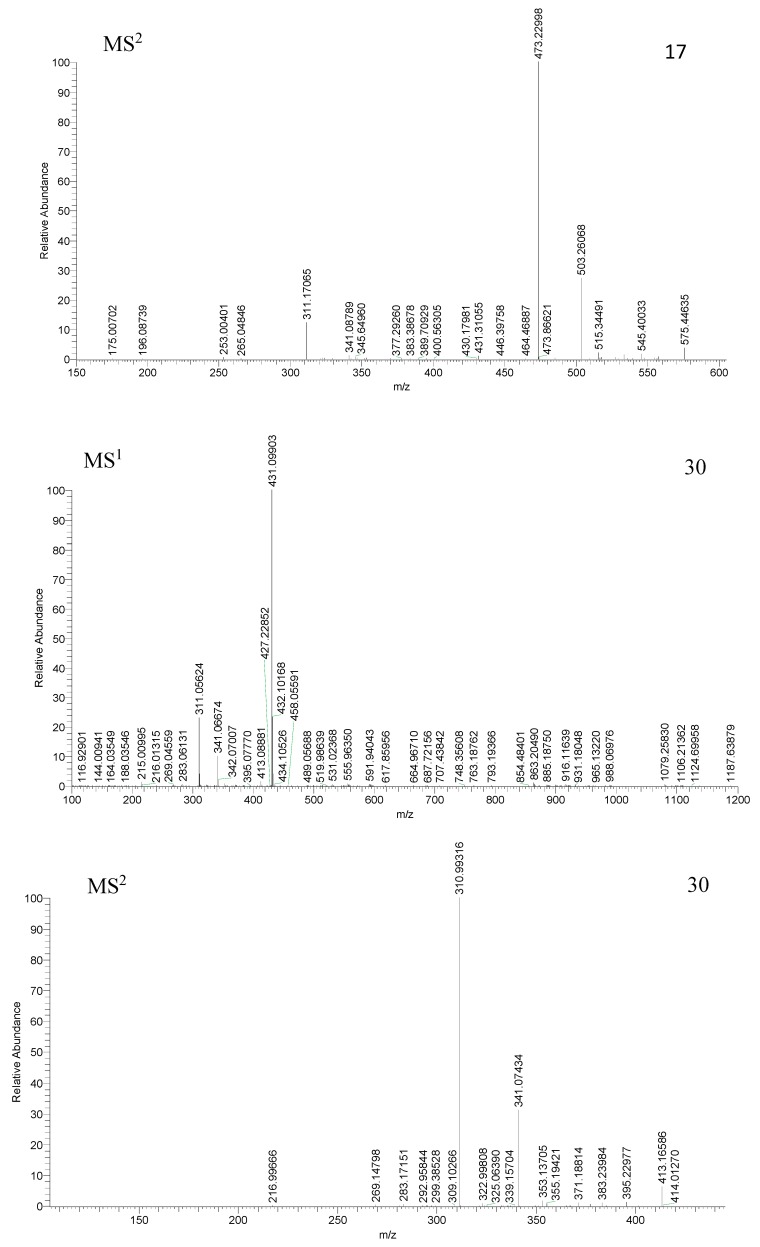

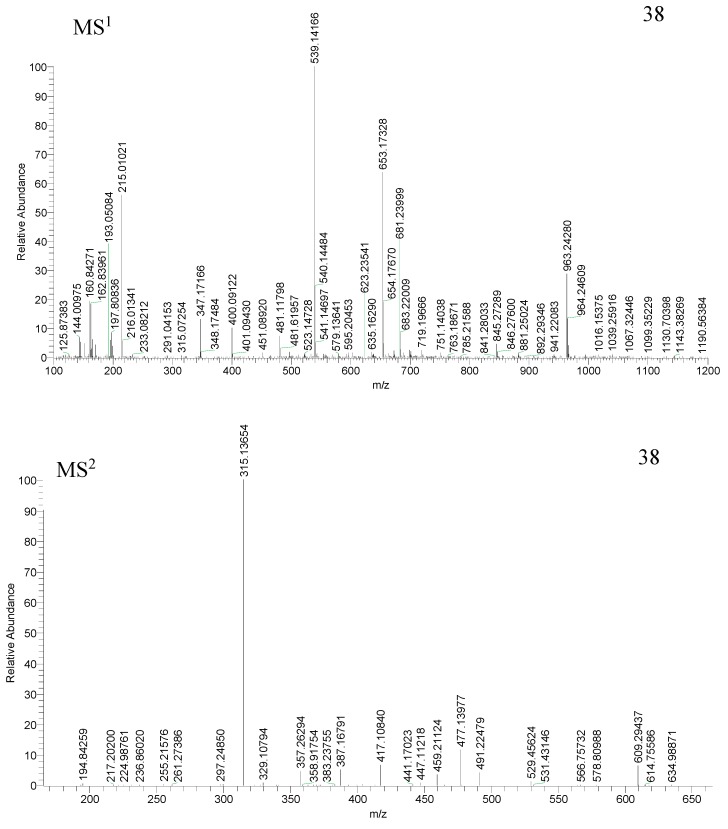
Spectra of ion fragments in MS^n^ analysis of isovitexin-2′′-*O*-B-d-glucosyle (**16**), saponarin (**17**), isovitexin, isopyrenine-7-*O*-glucosyle (**38**) in negative ion mode.

**Figure 8:**
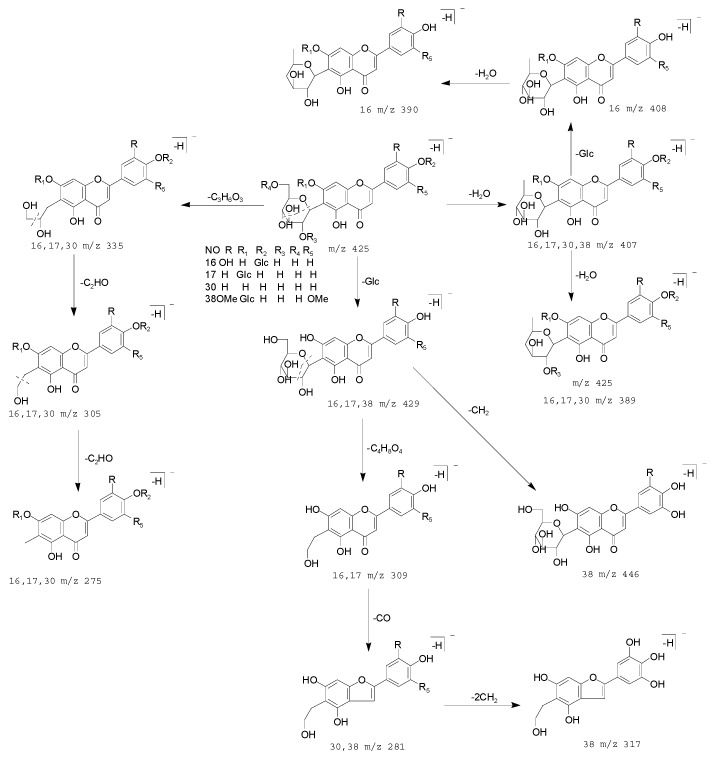
The proposed fragmentation pathway of saponarin, isovitexin-2′′-*O*-B-d-glucosyle, isovitexin, isopyrenine-7-*O*-glucosyle, 4′-*O*-B-d-glucospranosyl-2′’-*O*-[1-*O*-B-d-glucosyl-2,4,4-trihydroxy-(*E*)-cinnamoyl]-2′′-isoorientin.

Compounds 16, 17, 30 and 38 generated their [M − H]^−^ ions at *m*/*z* 609.14783 and 609.14764 (C_27_H_29_O_16_, mass error = 4.628 ppm and mass error = 4.316 ppm), *m*/*z* 593.15302 and 593.15277 (C_27_H_29_O_15_, mass error = 4.929 ppm and mass error = 4.507 ppm), *m*/*z* 431.09903 (C_21_H_19_O_10_, mass error = 4.075 ppm), *m*/*z* 653.17395 and *m*/*z* 653.17328 (C_29_H_33_O_17_, mass error = 2.724 ppm and mass error = 2.050 ppm), respectively. All of them produced [M − H − H_2_O]^−^ ions at *m*/*z* 591, *m*/*z* 575, *m*/*z* 413 and *m*/*z* 635, respectively. Compounds 16, 17 and 30 also generated the [M − H − 2H_2_O]^−^, [M − H − C_3_H_6_O_3_]^−^ and [M − H − C_4_H_8_O_4_]^−^ [47], ions at *m*/*z* 573, *m*/*z* 557, *m*/*z* 395, *m*/*z* 519, *m*/*z* 503, *m*/*z* 341, *m*/*z* 489, *m*/*z* 473, *m*/*z* 311. Compounds 16, 17 and 38 generated the [M − H − Glc]^−^ ions at *m*/*z* 447, *m*/*z* 431, *m*/*z* 491. Compounds 16 and 17 generated the [M − H − Glc − C_4_H_8_O_4_]^−^ ions at *m*/*z* 327, *m*/*z* 311. Moreover, compounds 16 generated ions at *m*/*z* 429 [M − H − Glc − H_2_O]^−^ and *m*/*z* 411 [M − H − Glc − 2H_2_O]^−^.Compound 30 ion generated ions at *m*/*z* 281 [M − H − C_5_H_10_O_5_]^−^ and *m*/*z* 283 [M − H − Glc − C_4_H_8_O_4_ − CO]^−^. In addition, compound 38 ion generated ions at *m*/*z* 477 [M − H − Glc − CH_2_]^−^, *m*/*z* 357 [M − H − Glc − C_4_H_8_O_4_ − CO]^−^ and *m*/*z* 315 [M − H − Glc − C_4_H_8_O_4_ − CO − 2CH_2_]^−^ [48]. The fragmentation pathways were consistent with deduced of compounds 16, 17, 30 and 38, which further proved the validity of the results (Figure 7 and Figure 8). Therefore, combined with bibliography data and fragmentation pathways, these four compounds were tentatively identified as isovitexin-2′′-*O*-B-d-glucosyle, saponarin, isovitexin, isopyrenine-7-*O*-glucosyle.

Compounds 25 and 28 produced their [M − H + HCOOH]^−^ ions at *m*/*z* 801.21179 and 801.21118 (C_34_H_41_O_22_, mass error = 3.391 ppm and mass error = 2.781 ppm), *m*/*z* 639.15826 and 639.15808401.10892 (C_28_H_31_O_17_, mass error = 2.684 ppm and mass error = 2.504), respectively. Both of them produced [M − H]^−^ and [M − H + HCOOH − Glc]^−^ ions at *m*/*z* 755, *m*/*z* 593, *m*/*z* 639 and *m*/*z* 477. Compound 25 generated a serial of ions at *m*/*z* 621 [M − H + HCOOH − Glc − H_2_O]^−^, *m*/*z* 477 [M − H + HCOOH − 2Glc]^−^, *m*/*z* 681 [M − H + HCOOH − C_4_H_8_O_4_]^−^ and *m*/*z* 635 [M − H − C_4_H_8_O_4_]^−^ [49]. Compound 28 could produce [M – H − Glc]^−^, [M − H + HCOOH − Glc − C_4_H_8_O_4_]^−^, [M − H − C_4_H_8_O_4_]^−^ and [M − H − C_5_H_10_O_5_]^−^ at *m*/*z* 431, *m*/*z* 357, *m*/*z* 311 and *m*/*z* 281, respectively. Therefore, according to the fragmentation pathways and literature data, compounds 25 and 28 were isovitexin-2′′-4′-*O*-B-d-glucosyle, isovitexin-2′′-*O*-B-d-glucosyle.

Compounds 27 and 36 yielded their [M + H]^+^ ions at *m*/*z* 449.10727 and 449.10684 (C_21_H_21_O_11_, mass error = 0.568 ppm and 0.998 ppm), *m*/*z* 493.13306 and 493.13287 (C_23_H_25_O_12_, mass error = 2.013 ppm and 2.398 ppm), respectively. Both of them generated [M + H − H_2_O]^+^ and ions [M + H − 2H_2_O]^+^ at *m*/*z* 431, *m*/*z* 475, *m*/*z* 413 and *m*/*z* 457, respectively. Compound 27 could generate a series of ions at *m*/*z* 395 [M + H − 3H_2_O]^+^, *m*/*z* 329 [M + H − C_4_H_8_O_4_]^+^ and *m*/*z* 299 [M + H − C_5_H_10_O_5_]^+^ [50,51]. Compound 36 displayed fragment ions at *m*/*z* 315, *m*/*z* 313 and *m*/*z* 195 corresponding to [M + H − C_10_H_10_O_3_]^+^, [M + H − C_5_H_10_O_5_ − HCHO]^+^ and [M + H − C_5_H_10_O_5_ − C_10_H_10_O_3_]^+^ respectively. By comparing with the literature data, compounds 27 and 36 were tentatively annotated as isoorientin and 6-*C*-B-d-glucospranosyltricine (isopyrenine).

Compounds 39 and 46 produced their [M − H + CH_3_COOH]^−^ ions at *m*/*z* 683.18146 (C_30_H_35_O_18_, mass error = 0.484 ppm) and *m*/*z* 521.12724 (C_24_H_25_O_13_, mass error = 1.653 ppm). Both of the deprotonated molecular ions yield [M − H + CH_3_COOH − H_2_O]^−^ at *m*/*z* 503 and *m*/*z* 665. Compound 39 produced their [M − H + CH_3_COOH − 2H_2_O]^−^, [M − H − CH_2_]^−^, [M − H − Glc − CO]^−^, [M − H − Glc − C_5_H_10_O_5_]^−^ and [M − H − Glc − C_5_H_10_O_5_ − CH_2_]^−^ ions at *m*/*z* 647, *m*/*z* 609, *m*/*z* 433, *m*/*z* 311 and *m*/*z* 297 [52]. Compound 46 produced their [M − H + CH_3_COOH − CO_2_]^−^, [M − H − C_4_H_8_O_4_]^−^, [M − H − C_5_H_10_O_5_ − CH_2_]^−^ and [M − H − C_5_H_10_O_5_ − C_9_H_8_O_2_]^−^ ions at *m*/*z* 477, *m*/*z* 341, *m*/*z* 297 and *m*/*z* 163. According to the literature data, compounds 39 and 46 were tentatively annotated as ioscoparine-7-*O*-B-d-glucosyle and isoscoparine.

Compound 42 produced the [M − H + CH_3_COOH]^−^ ion at *m*/*z* 1009.24139 (C_44_H_49_O_27_, mass error = 4.144 ppm). It generated a serial of ions at *m*/*z* 991 [M − H + CH_3_COOH − H_2_O]^−^, *m*/*z* 973 [M − H + CH_3_COOH − 2H_2_O]^−^, *m*/*z* 949 [M − H]^−^, *m*/*z* 931 [M − H − H_2_O]^−^, *m*/*z* 847 [M − H + CH_3_COOH − Glc]^−^, *m*/*z* 846 [M − H + CH_3_COOH − Glc − H]^−^, *m*/*z* 489 [M − H − C_15_H_16_O_9_ − C_4_H_8_O_4_]^−^ and *m*/*z* 489 [M − H + CH_3_COOH − C_15_H_16_O_9_ − C_5_H_10_O_5_]^−^. According to the literature data, compound 42 was ascertained as 4′-*O*-B-d-glucospranosyl-2′′-*O*-[1-*O*-B-d-glucosyl-2,4,4-trihydroxy-(*E*)-cinnamoyl]-2′′-isoorientin.

#### 2.1.3. Structural Characterization and Identification of Xanthones

Compound 3 showed [M − H + HCOOH]^−^ ion at *m*/*z* 449.10971 and 449.40989 (C_21_H_21_O_11_, mass error = 4.169 ppm and 4.569 ppm). Its ESI-MS^2^ base peak ion at *m*/*z* 359 was generated by losing C_3_H_6_O_3_. In addition, the major ions at *m*/*z* 329 and *m*/*z* 299 were produced by neutral loss of C_4_H_10_O_4_ and C_5_H_10_O_5_, respectively. Moreover, the molecular ion also yielded ions at *m*/*z* 283 [M − H − C_4_H_10_O_4_]^−^, *m*/*z* 253[M − H − C_5_H_10_O_5_]^−^ and *m*/*z* 179 [Glu − H]^−^ [53]. By comparing with the literature data, compound 3 was tentatively identified as 2-*C*-β-d-glucospranosyl-glucopyranosyl-1-hydroxy-7-methoxyxanthone.

Compounds 7 and 24 produced its [M − H + HCOOH]^−^ ion at *m*/*z* 451.14664 (C_20_H_19_O_12_, mass error = 4.850 ppm), *m*/*z* 377.08716 and 377.08698 (C_18_H_17_O_9_, mass error = 1.197 ppm and 0.720 ppm). Both of the deprotonated molecular ions at *m*/*z* 405 and 331 [M − H]^−^. Compound 7 generated a series of ions *m*/*z* 433 [M − H + HCOOH − H_2_O]^−^, *m*/*z* 415 [M − H + HCOOH − 2H_2_O]^−^, *m*/*z* 243 [M − H − Glc]^−^ and *m*/*z* 269 [M − H − C_7_H_4_O_3_]^−^. Compound 24 generated ions *m*/*z* 347 [M − H + HCOOH − HCHO]^−^, *m*/*z* 197 [M − H − C_8_H_6_O_2_]^−^, *m*/*z* 119 [M − H − C_9_H_10_O_4_]^−^, *m*/*z* 153 [M − H + HCOOH − HCHO − C_8_H_6_O_2_]^−^ and *m*/*z* 179 [M − H − C_7_H_6_O_2_ − HCHO]^−^ [54]. Therefore, compounds 7 and 24 were tentatively identified as 2-*O*-β-d-glucospranosyl-1,6-dihydroxyxanthone and 1-hydroxy-2,3,4,7-tetramethoxy xanthone.

Compounds 9 and 29 produced [M − H + CH_3_COOH]^−^ ions at *m*/*z* 375.10927 (C_19_H_19_O_8_, mass error = 4.895 ppm), *m*/*z* 523.14722 and 523.14703 (C_24_H_27_O_13_, mass error = 4.975 ppm and 4.612 ppm). Both of the deprotonated molecular ion yield [M − H + CH_3_COOH − C_9_H_8_O_4_]^−^ at *m*/*z* 195 and at *m*/*z* 343. Compound 9 produced a serial of ions at *m*/*z* 357 [M – H + CH_3_COOH − H_2_O]^−^, *m*/*z* 287 [M − H − CO]^−^, *m*/*z* 255 [M − H − 2HCHO]^−^, *m*/*z* 179 [M − H − C_8_H_8_O_2_]^−^, *m*/*z* 151 [M − H − C_9_H_8_O_3_]^−^ and *m*/*z* 121 [M − H − C_9_H_8_O_3_ − HCHO]^−^. Compound 29 generated [M − H + CH_3_COOH − 2H_2_O]^−^, [M − H + CH_3_COOH − 4H_2_O]^−^, [M − H + CH_3_COOH − Glc]^−^, [M − H + CH_3_COOH − C_8_H_8_O_3_]^−^, [M − H + CH_3_COOH − C_9_H_8_O_3_]^−^, [M − H − C_9_H_8_O_3_]^−^, [M − H – Glc − 2HCOH]^−^ and [M − H – Glc − C_8_H_8_O_2_]^−^ at *m*/*z* 487, *m*/*z* 451, *m*/*z* 361, *m*/*z* 371, *m*/*z* 359, *m*/*z* 299, *m*/*z* 241 and *m*/*z* 165, respectively. According to the literature data, compounds 9 and 29 were tentatively determined as tetramethoxy-1,3,7,8-xanthone and glucosyl-1-decussatin.

Compounds 10, 13 and 54 generated their [M − H]^−^ ions at *m*/*z* 581.14722 (C_26_H_29_O_15_, mass error = 4.950 ppm), *m*/*z* 449.10965 (C_21_H_21_O_11_, mass error = 4.035 ppm), *m*/*z* 595.16437 (C_27_H_31_O_15_, mass error = 2.313 ppm), respectively. Compounds 10 and 54 produced [M − H − CO − H_2_O ]^−^ ion at *m*/*z* 534 and *m*/*z* 549. Compounds 13 and 54 also generated the [M − H − H_2_O]^−^ and [M − H − 2H_2_O]^−^ ions at *m*/*z* 431, *m*/*z* 577, *m*/*z* 413 and *m*/*z* 559. Compound 10 generated [M − H − 2HCHO]^−^, [M − H − CO − 2H_2_O]^−^, [M − H − CO − 3H_2_O]^−^, [M − H − CO − Primeverosyl]^−^ and [M − H − HCHO − Primeverosyl]^−^, ions at *m*/*z* 521, *m*/*z* 517, *m*/*z* 499, *m*/*z* 249 and *m*/*z* 247. Compound 13 generated the [M − H − CO − H_2_O]^−^, [M − H − C_7_H_6_O_2_]^−^, [M − H − C_8_H_6_O_4_]^−^ and [M − H − Glc − CO − H_2_O]^−^, ions at *m*/*z* 403, *m*/*z* 327, *m*/*z* 283 and *m*/*z* 241. Moreover, compound 54 generated ions at *m*/*z* 495 [M − H − CO − 4H_2_O]^−^, *m*/*z* 477 [M − H − CO − 5H_2_O]^−^, *m*/*z* 417 [M − H − CO − C_5_H_8_O_5_]^−^, *m*/*z* 415 [M − H − C_9_H_8_O_4_]^−^ and *m*/*z* 279 [M − H − C_5_H_8_O_4_ − C_8_H_8_O_2_]^−^. Therefore, combined with bibliography data and fragmentation pathways, these three compounds were tentatively identified as gentiabavaroside, glucosyl-1-gentiacaulein, primeverosyl-1-decussatin.

Compound 18 produced its [M − H + HCOOH]^−^ ion at *m*/*z* 481.09833 (C_21_H_21_O_13_, mass error = 1.378 ppm). The [M − H + HCOOH]^−^ ion of compound 18 produced the aglycone ion at *m*/*z* 273 in the MS^2^ spectrum, which originated from the neutral loss of an glucose moiety (162 Da). It also generated a series of ions at *m*/*z* 435 [M − H]^−^, *m*/*z* 445 [M − H + HCOOH − 2H_2_O]^−^, *m*/*z* 403 [M − H − CH_2_ − H_2_O]^−^, *m*/*z* 359 [M − H − C_7_H_6_O_2_ ]^−^ and *m*/*z* 241 [M − H − Glc − 2H_2_O]^−^. Therefore, it was tentatively identified as glucosyl-8-swertianin.

**Figure 9:**
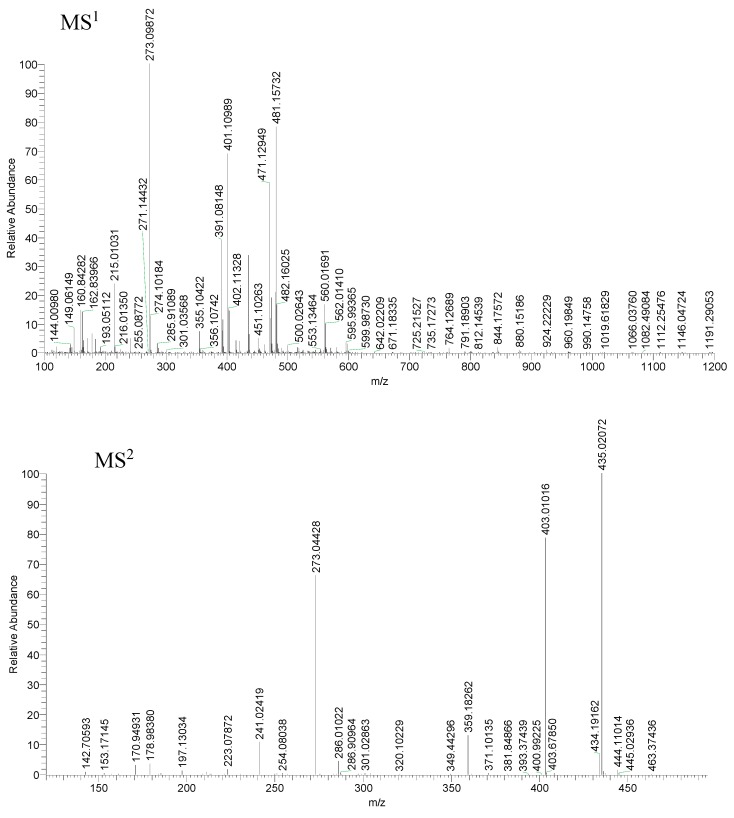
Spectra of ion fragments in MS^n^ analysis of mangiferin in negative ion modea.

**Figure 10:**
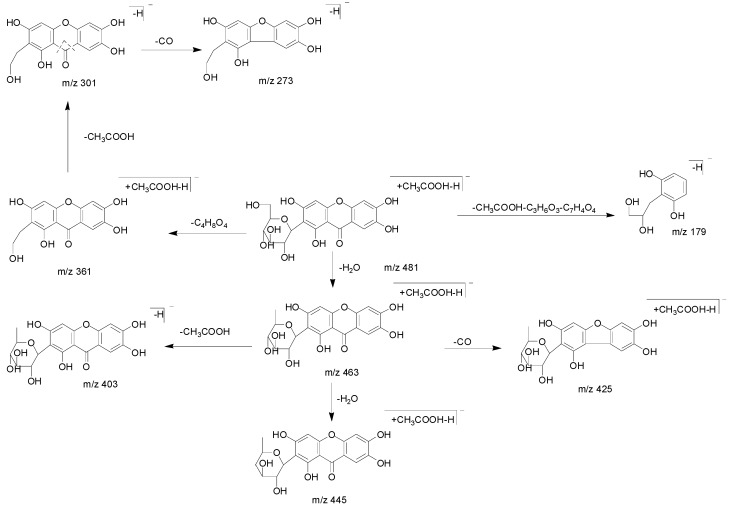
The proposed fragmentation pathway of mangiferin.

Compound 19 produced the [M − H + CH_3_COOH]^−^ ion at *m*/*z* 481.09711 (C_21_H_21_O_13_, mass error = 1.158 ppm). It generated a serial of ions at *m*/*z* 463 [M − H + CH_3_COOH − H_2_O]^−^, *m*/*z* 445 [M − H + CH_3_COOH − 2H_2_O]^−^, *m*/*z* 435 [M − H + CH_3_COOH − H_2_O − CO]^−^, *m*/*z* 403 [M − H − H_2_O]^−^, *m*/*z* 361 M − H + CH_3_COOH − C_4_H_8_O_4_]^−^, *m*/*z* 301 [M − H − C_4_H_8_O_4_]^−^, *m*/*z* 273 [M − H − C_4_H_8_O_4_ − CO]^−^ and *m*/*z* 179 [M − H − C_4_H_8_O_4_ − C_7_H_4_O_4_]^−^. The fragmentation pathway was consistent with deduced of compound 19, which further proved the validity of the results (Figure 9 and Figure 10). Compared with the t_R_ values and mass spectra with the reference standard, compound 19 was ascertained as mangiferin [55,56,57].

Compound 23 produced the [M − H + CH_3_COOH]^−^ ion at *m*/*z* 377.08704and 377.08734 (C_18_H_17_O_9_, mass error = 0.879 ppm and 1.657 ppm). It generated [M − H + CH_3_COOH − CH_3_]^−^, [M − H + CH_3_COOH − H_2_O]^−^, [M − H + CH_3_COOH − HCHO]^−^, [M − H + CH_3_COOH − C_9_H_8_O_4_]^−^, [M − H − C_7_H_6_O_3_]^−^ and [M − H − C_7_H_6_O_3_ − H_2_O − CO_2_]^−^ ions at *m*/*z* 362, *m*/*z* 359, *m*/*z* 347, *m*/*z* 197, *m*/*z* 179 and *m*/*z* 153. Compound 23 was ascertained as 2-methylcorymbiferin.

Compounds 32, 33 and 44 gave their [M + NH_4_]^+^ ions at *m*/*z* 570.18396 (C_25_H_28_O_14_N, mass error = 3.909 ppm), *m*/*z* 570.18060 and 570.18390 (C_25_H_32_O_14_N, mass error = 1.984 ppm and 3.804 ppm), *m*/*z* 570.18390 and 570.18054 (C_25_H_32_O_14_, mass error = 3.804 ppm and 2.089 ppm), so they were isomers. They all produced the [M + H − CO]^+^ and ions at *m*/*z* 525. Besides, compounds 32 and 33 gave [M + H − CO − H_2_O]^+^ ion at *m*/*z* 507. Compounds 32 and 44 could generate [M + H]^+^ ion at *m*/*z* 553. In addition, compound 32 could generate [M − C_7_H_6_O_2_ − C_5_H_8_O_4_ − CH_3_]^+^, [M + H − C_7_H_6_O − C_5_H_8_O_4_]^+^ and [M + H − C_5_H_8_O_5_ − C_8_H_6_O_3_]^+^ at *m*/*z* 283, *m*/*z* 287 and *m*/*z* 253. Compound 33 generated [M + H − CO − 2H_2_O]^+^, [M + H − C_5_H_8_O_4_ − C_7_H_6_O_3_]^+^ and [M + H − C_7_H_6_O_3_ − C_5_H_8_O_4_]^+^ at *m*/*z* 489, *m*/*z* 283 and *m*/*z* 265. Compound 44 generated [M + H − C_5_H_8_O_4_ − C_7_H_4_O_2_]^+^, [M + H − C_5_H_8_O_4_ − C_6_H_4_O_2_ − CO]^+^, [M + H − C_11_H_19_O_9_]^+^, [M + H − C_11_H_19_O_9_ − CO]^+^ and [M + H − C_5_H_8_O_4_ − C_7_H_4_O_2_ − CO]^+^ at *m*/*z* 283, *m*/*z* 265, *m*/*z* 259, *m*/*z* 229 and *m*/*z* 219. Therefore, compounds 32, 33 and 44 were tentatively ascertained as gentioside, 1-hydroxy-2-methoxy-7-*O*-primeveroylxanthone and 7-hydroxy-2-methoxy-1-*O*-primeveroyl xanthone.

Compounds 34 and 35 produced [M + K]^+^ ions at *m*/*z* 298.99619 (C_13_H_8_O_6_K, mass error = 0.944 ppm) and *m*/*z* 298.99518 (C_13_H_8_O_6_K, mass error = 0.565 ppm). Compounds 34 and 35 produced a serial of ions at *m*/*z* 299 [M + K]^+^, *m*/*z* 271 [M + K − CO]^+^, *m*/*z* 255 [M + K − CO − H_2_O]^+^ and *m*/*z* 155 [M + K − C_6_H_8_O_2_ − 2H_2_O]^+^ and *m*/*z* 136 [M − C_6_H_4_O_3_ + H]^+^ [58]. According to the literature data, compound 34 and 35 were tentatively determined as desmethylbellidifolin and norswertianin.

Compounds 37 and 43 gave their [M + NH_4_]^+^ ions at *m*/*z* 586.17798 and 586.17841 (C_25_H_32_O_15_N, mass error = 2.277 and 3.070 ppm), *m*/*z* 444.15210 (C_19_H_26_O_11_N, mass error = 4.654 ppm). Both of them produced [M + H]^+^ and [M + H − CO]^+^ ions at *m*/*z* 569, *m*/*z* 427, *m*/*z* 541 and *m*/*z* 399. Compound 37 showed [M + H − H_2_O]^+^, [M + H − HCHO]^+^, [M + H − C_5_H_8_O_4_ − C_7_H_6_O_3_]^+^ and [M + H − C_5_H_8_O_4_ − C_8_H_6_O_4_ − CO]^+^ ions at *m*/*z* 551, *m*/*z* 539, *m*/*z* 299 and *m*/*z* 243. Compound 43 generated [M + NH_4_ − H_2_O]^+^, [M + H − CO − H_2_O]^+^. [M + H − Glc − CO]^+^ and [M + H − Glc − CO − H_2_O]^+^ at *m*/*z* 426, *m*/*z* 381, *m*/*z* 237 and *m*/*z* 219. Compounds 37 and 43 were tentatively determined as gentiakochianoside and 1,3,4-trihydroxy-8-β-d-glucospranosyl-5,6,7,8-tetrahydroxanthone.

Compound 40 showed [M − H + HCOOH]^−^ ion at *m*/*z* 511.10571 and 511.10580 (C_21_H_21_O_11_, mass error = 4.934 and 4.758 ppm). In ESI-MS^2^, its ions at *m*/*z* 493 [M − H + HCOOH − H_2_O]^−^, *m*/*z* 475 [M − H + HCOOH − 2H_2_O]^−^, *m*/*z* 467 [M − H + HCOOH − CO_2_]^−^, *m*/*z* 465 [M − H]^−^, *m*/*z* 349 [M − H + HCOOH − Glc]^−^, *m*/*z* 305 [M − H + HCOOH − Glc − CO_2_]^−^, *m*/*z* 297 [M − H − H_2_O − C_8_H_6_O_3_]^−^ and *m*/*z* 153 [M − H − Glc − C_8_H_6_O_3_]^−^. By comparing with the literature data, compound 40 was tentatively identified as 1-*O*-glucosyl corymbiferi.

Compound 47 generated its [M + Na]^+^ ion at *m*/*z* 443.09613 (C_20_H_20_O_10_Na, mass error = 2.848 ppm). It produced the [M + Na − H_2_O]^+^, [M + Na − 2H_2_O]^+^and [M + Na − 3H_2_O]^+^ ions at *m*/*z* 425, *m*/*z* 407 and *m*/*z* 389. The [M + Na]^+^ ion produced the ions at *m*/*z* 281, *m*/*z* 263 and *m*/*z* 247 in the MS^2^ spectrum, which originated from the neutral loss of glucose, H_2_O and CH_4_. Therefore, compound 47 was tentatively deduced as 8-*O*-glucosyl bellidifolin.

#### 2.1.4. Structural Characterization and Identification of Other

Compound 53 produced the [M − H]^−^ ion at *m*/*z* 455.35406 and 455.35413 (mass error = 2.784 and 2.812 ppm) (C_30_H_47_O_3_). The molecular ions yield [M − H − H_2_O]^−^, [M − H − CO_2_]^−^, [M − H − HCOOH]^−^, [M − H − C_15_H_26_O]^−^, [M − H − C_15_H_22_O_2_]^−^, [M − H − C_15_H_26_O − CO_2_]^−^ and [M − H − C_15_H_26_O − CO_2_ − CH_2_]^−^ at *m*/*z* 437, *m*/*z* 411, *m*/*z* 409, *m*/*z* 233, *m*/*z* 221, *m*/*z* 189 and *m*/*z* 175. According to the literature data, compound 53 was tentatively deduced as oleanolic acid.

## 3. Materials and Methods

### 3.1. Materials and Chemicals

*Gentiana radix* was purchased from Anguo herbs market (Anguo, Hebei, China) and was identified as the dry root and rhizome of *Gentiana scabra* Bge. by professor Li Feng of Liaoning University of Traditional Chinese Medicine. Wine-processed *Gentiana radix* is made by reference to the Chinese pharmacopoeia (2015). Gentiopicroside, swertiamarin, sweroside, loganin, isovitexin, and mangiferin were purchased from Jiangsu Yongjian Pharmaceutical Technology CO., Ltd. (Taizhou, Jiangsu, China). Formic acid, methanol and acetonitrile (HPLC grade) were purchased from Fisher Scientific (Fair Lawn, NJ, USA). Ultrapure water was purchased from Hangzhou Wahaha Group Co., Ltd. (Hangzhou, Zhejiang, China). Yellow rice wine was purchased from Zhejiang Tapai Shaoxing Liquor Co., Ltd. (Shaoxing, Zhejiang, China). Preparation of wine-processed *Gentiana radix:* After diluting the yellow rice wine with water, mix it with raw *Gentiana radix*. (raw *Gentiana radix*: yellow rice wine: water = 10:1:1.5). After moistening for 30 min, stir it in a stir-frying container and keep it stir-frying for 4 min at 100 °C, stirring 100 times per minute. When the raw *Gentiana radix* is dark yellow on the surface and slightly fragrant with wine, take it out and cool it, resulting in wine-processed *Gentiana radix*.

### 3.2. Sample and Standards Preparation

The standard solutions of gentiopicroside, swertiamarin, sweroside, loganin, isovitexin, and mangiferinwere prepared in methanol at appropriate concentrations. The powders of crude and wine-processed *Gentiana radix* were weighed precisely (0.50 g); the powders were placed into 20 mL of methanol and the quality was recorded, and then ultrasonically extracted at room temperature for 0.5 h. The extracts were cooled to room temperature and filtered to a 25-mL volumetric flask, then methanol supplemented to 25 mL. Methanol extracts were filtered through a 0.22-µm membrane for analysis. All of the solutions were stored at 4 °C and brought to room temperature before analysis.

### 3.3. Instrumentation and Condition

UHPLC analysis was performed on DIONEX Ultimate 3000 UHPLC system (Thermo Fisher Scientific, Waltham, MA, USA) with a binary pump and an autosampler. Samples were separated on a BEH C18 (2.1 × 100 mm, 1.7 µm, Acquity Corporation, Ireland, MA, USA) at room temperature. The mobile phase consisted of 0.1% (*v*/*v*) formic acid and acetonitrile (B). A gradient program was adopted as follows: 0–2 min, 2% B; 2–20 min, 2–22% B; 20–24 min, 22–80%; 24–26min, 80% B; 26–27 min, 80–2% B; 27–30 min, 2% B. The flow rate was set as 0.3 mL/min.

A LTQ-Orbitrap XL mass spectrometer (Thermo Scientific, Bremen, Germany) was connected to the UHPLC system via an electrospray ionization (ESI) interface. The effluent was introduced into the ESI source in a post-column splitting ratio of 1:4. The analysis was performed in both negative and positive ion mode with a mass range of *m*/*z* 100–200.

The analysis was performed in both negative and positive ion mode with a mass range of *m*/*z* 100–1500. The optimized ESI parameters in negative ion mode were set as follows: capillary temperature of 350 °C; sheath gas (nitrogen) flow of 30 arb.; auxiliary gas (nitrogen) flow of 10 arb.; source voltage of 3.0 kV; capillary voltage of −35 V; tube lens voltage of −110 V. The capillary voltage was 25 V, source voltage of 4.0 kV and tube lens voltage was 110 V in positive ion mode; and other parameters were same as those of negative ion mode. The resolution of the orbitrap mass analyzer was set at 30,000. The isolation width was 2 amu, and the normalized collision energy (CE) was set to 35%. Collision-induced dissociation (CID) was conducted in LTQ with an activation q of 0.25 and activation time of 30 ms. All instruments were controlled by the Xcalibur data system, and the data acquisition was carried out by analyst software Xcalibur (version 2.1) from Thermo Electron Corp (Waltham, MA, USA).

## 4. Conclusions

In this study, an effective and sensitive analytical method by UHPLC-LTQ-Orbitrap-MS^n^ was established for systematically characterizing constituents in crude and wine-processed *Gentiana radix*. By this method, the structure of constituents could be identified and the peak area of constituents could be determined. The wine-processing would change the structure of chemical constituents of *Gentiana scabra*, which resulted in partial loss and abscission of unstable chemical groups (hydroxyl, glucosyle, carboxyl, etc.), and carbon–carbon double bond would be displaced or broken in the structure of some constituents.

The results showed that there were 10 characteristic components in the crude *Gentiana radix*, which were 2-*O*-β-d-glucospranosyl-1,6-dihydroxyxanthone, tetramethoxy-1,3,7,8-xanthone, glucosyl-8-swertianin, mangiferin, 2′-feruloy loganin, isoscoparine-7-*O*-B-d-glucosyle, 4′-*O*-B-d-glucospranosyl-2′′-*O*-[1-*O*-B-d-glucosyl-2,4,4-trihydroxy-(*E*)-cinnamoyl]-2′-isoorientin, 8-*O*-glucosyl bellidifolin, gentianaside, scabran G4. And 8 characteristic constituents in wine-processed *Gentiana radix*, which are gentiabavaroside, glucosyl-1-gentiacaulein, gentioside, desmethylfoidilin, norswertianin, 1,3,4-trihydroxy-8-β-d-glucospranosyl-5,6,7,8-tetrahydroxanthone, isoscoparine, primeverosyl-1-decussatin.

Since the sampling amounts of crude and wine-processed *Gentiana radix* are the same, by comparing the peak area of their chemical constituents, it can be seen that the contents of some chemical constituents change in *Gentiana radix* after being wine-processed. Among the constituents, 2-*C*-β-d-glucospranosyl-glucopyranosyl-1-hydroxy-7-methoxyxanthone, methyl (1*S*)-1-(d-glucopyranosyloxy)-6,7-dihydroxy-7-methyl-1,6,7,7a-tetrahydrocyclopenta[c]pyran-4-carboxylate, glucosyl-1-decussatin, gentiakochianoside, 1-*O*-glucosyl corymbiferin, macrophylloside A and oleanolic acid increased significantly after being wine-processed. While 2-methylcorymbiferin, 1-hydroxy-2,3,4,7-tetramethoxy xanthone, 1-hydroxy-2-methoxy-7-*O*-primeveroylxanthone and morroniside decreased significantly after being wine-processed.

By comparing the structure and peak area of the constituents, we could see that there are differences between crude and wine-processed *Gentiana radix,* which showed wine-processing can change the structure and contents of some constituents in *Gentiana radix*. The result of this paper can be used to explain the processing principles of *Gentiana radix* to some extent.

## Figures and Tables

**Table 1:** Identification of chemical constituents of crude and wine-processed *Gentiana radix* by UHPLC-LTQ-Orbitrap.

No.	t_R_/min		Identification	Empirical Formula	Proposal Ions	Theoretical Mass *m*/*z*	Experimental Mass *m*/*z*		Mass Error (ppm)		MS^2^ Data (Measured)		Chromatographic Peak Area Ratio (Crude: Wine)
	Crude	Wine					Crude	Wine	Crude	Wine	Crude	Wine	
1	7.50	7.51	Loganetin ^a,b^ [15]	C_11_H_17_O_5_	[M + H]^+^	229.10705	229.10652	229.10654	2.314	2.227	211(100), 193(2.73), 197(0.95), 169(1.45), 179(2.72), 151(1.47), 161(0.09), 133(0.31)	211(100), 193(0.42), 197(1), 169(0.31), 179(1.21), 151(2.35), 161(0.22)	1.11
2	7.70	7.71	Eustomoside ^a,b^ [16]	C_16_H_23_O_11_	[M + H]^+^	391.12349	391.12317	391.12280	0.813	1.759	373(0.35), 229(100), 211(37.5), 193(0.39), 125(0.98)	373(0.35), 229(100), 211(37.5), 193(0.39), 125(0.98)	0.91
3	9.75	9.74	2-*C*-β-d-glucospranosyl-glucopyranosyl-1-hydroxy-7-methoxyxanthone ^a,b^ [17]	C_21_H_21_O_11_	[M − H + HCOOH]^−^	449.10784	449.10971	449.40989	4.169	4.569	359(100), 329(0.13), 283(0.47), 327(0.06), 241(43.13), 179(17.1)	359(100), 329(0.22), 283(0.33), 253(0.04), 299(0.15), 179(27.08)	0.32
4	9.88	9.90	Methyl (1*S*)-1-(d-glucopyranosyloxy)-6,7-dihydroxy-7-methyl-1,6,7,7a-tetrahydrocyclopenta[c]pyran-4-carboxylate ^a,b^ [18]	C_17_H_25_O_11_	[M + H]^+^	405.13914	405.13898	405.13864	0.390	1.229	387(1.33), 373(0.06), 345(0.16), 243(100), 225(0.26), 211(38.42), 193(0.27), 175(0.35), 165(0.51)	387(0.24), 373(0.09), 345(0.06), 243(100), 225(0.23), 211(38.3), 193(0.73), 175(0.1), 165(0.25)	0.75
5	10.17	10.20	Loganic acid ^a,b^	C_16_H_23_O_10_	[M − H]^−^	375.12857	375.13019	375.13022	4.310	4.390	213(100), 195(0.27), 169(17.87), 113(1.83), 151(3.61), 125(2.48)	213(100), 195(0.16), 169(18.11), 113(1.94), 151(3.66), 125(2.69)	0.91
6	10.29	10.31	1-*O*-d-glucopyransylampexine ^a,b^ [19]	C_16_H_26_O_9_Na	[M + Na]^+^	385.14690	385.14673	385.14664	0.450	0.684	367(0.46), 355(100), 357(0.02), 223(4.06), 205(0.13),	367(0.41), 355(100), 357(0.05), 339(0.13), 223(4.06), 205(0.04),	1.11
7	10.38	-	2-*O*-β-d-glucospranosyl-1,6-dihydroxyxanthone ^a^ [17]	C_20_H_19_O_12_	[M − H + HCOOH]^−^	451.08710	451.14664	-	4.850		405(100), 433(0.57), 415(0.63), 243(29.91), 269(0.28)	-	-
8	11.02	11.07	6′-*O*-β-d-glucosyl swertiamarin ^a,b^ [20]	C_23_H_33_O_17_	[M − H + HCOOH]^−^	581.17123	581.17352	581.17328	3.948	3.535	563(1.04), 545(4.78), 535(100), 517(17.57), 499(1.93), 341(36.38), 323(15.84), 193(1.11)	563(0.56), 545(3.64), 535(100), 517(25.37), 499(1.23), 341(29.58), 323(14), 193(0.96)	1.11
9	11.07	-	Tetramethoxy-1,3,7,8-xanthone ^a^ [21]	C_19_H_19_O_8_	[M − H + CH_3_COOH]^−^	375.10744	375.10927	-	4.895	-	357(2.16), 287(0.87), 255(0.71), 195(100), 179(5.46), 151(84.67), 121(22.05)	-	-
10	-	11.10	Gentiabavaroside ^b^ [21]	C_26_H_29_O_15_	[M − H]^−^	581.15010	-	581.14722	-	4.950		535(100), 521(1.15), 517(31.95), 499(4.9), 521(1.15), 249(0.4), 247(1.44)	-
11	11.63	11.65	Secologano ^a,b^ [22]	C_17_H_25_O_10_	[M − H]^−^	389.14422	389.14285	389.14258	3.529	4.223	371(4.98), 345(100), 209(30.88), 191(0.33), 163(0.35), 149(1.17)	371(3.63), 345(100), 311(0.2), 209(33.11), 191(0.44), 163(0.37), 149(1.9)	0.91
12	11.65	11.68	Kingiside ^a,b^ [23]	C_17_H_25_O_11_	[M + H]^+^	405.13914	405.13895	405.13858	0.188	0.558	387(0.07), 369(0.04), 243(100), 211(34.33), 207(0.02), 193(0.24), 183(0.13), 165(0.44)	387(0.07), 369(0.04), 243(100), 211(37.23), 207(0.01), 193(0.25), 183(0.13), 165(0.32)	0.86
13	-	11.69	Glucosyl-1-gentiacaulein ^b^ [21]	C_21_H_21_O_11_	[M − H]^−^	449.10784	-	449.10965	-	4.035	-	431(0.52), 403(4.87), 413(0.06), 241(100), 327(0.06), 283(0.06), 165(1.89)	-
14	11.80	11.82	Swertiamarin ^a,b,^*	C_17_H_23_O_12_	[M − H + HCOOH]^−^	419.11840	419.11990	419.11984	3.573	3.430	373(4.23), 355(12.27), 211(3.06), 193(0.1), 179(100), 161(5.74), 149(1.53)	373(4.22), 355(12.04), 211(2.43), 193(0.11), 179(100), 161(6.37), 149(1.6)	0.87
15	11.87	11.90	6′-*O*-β-d-glucosyl gentiopicroside ^a,b^ [24]	C_23_H_31_O_16_	[M − H + HCOOH]^−^	563.16066	563.16193	563.16144	2.168	2.048	517(31.74), 355(0.16), 499(1.98), 341(100), 323(13.4), 327(0.29), 305(1.5), 309(0.26), 193(1.63), 179(46.29), 165(0.03)	517(38.51), 355(0.05), 499(2.88), 341(100), 323(16), 305(1.26), 309(0.1), 193(3.28), 179(45.22), 175(0.14), 165(0.35)	0.91
16	12.51	12.53	Isoorientin-4′-*O-*β-d-glucosyle ^a,b^ [25]	C_27_H_29_O_16_	[M − H]^−^	609.14501	609.14783	609.14764	4.628	4.316	591(0.19), 573(0.21), 519(2.06), 447(100), 489(4.3), 327(5.65), 429(1.63), 411(0.45)	591(0.29), 573(0.14), 519(1.56), 447(100), 489(5.61), 327(5.35), 429(0.66), 411(0.07)	0.9
17	12.59	12.59	Saponarin ^a,b^ [26]	C_27_H_29_O_15_	[M − H]^−^	593.15010	593.15302	593.15277	4.929	4.507	575(3.67), 557(0.81), 503(27.18), 473(100), 431(0.98), 311(12.43)	575(4.38), 557(1.49), 503(26.83), 473(100), 431(1.1), 311(9.15)	1.17
18	12.79	-	Glucosyl-8-swertianin ^a^ [21]	C_21_H_21_O_13_	[M – H + HCOOH]^−^	481.09767	481.09833	-	1.378	-	445(0.28), 435(100), 273(49.56), 241(10.58), 403(69.94), 359(17.44)	-	-
19	12.86	-	Mangiferin ^a,^*	C_21_H_21_O_13_	[M − H + CH_3_COOH]^−^	481.09767	481.09711	-	1.158	-	463(0.13), 445(0.28), 435(100), 361(0.05), 301(0.34), 403(0.53), 273(66.13), 179(3.46)	-	-
20	12.96	12.98	Gentiopicroside ^a,b,^*	C_17_H_21_O_11_	[M − H + HCOOH]^−^	401.10784	401.10907	401.10892	1.232	1.082	355(78.18), 193(9.39), 179(100), 175(0.48), 149(14.29), 165(1.54)	355(78.98), 193(11.62), 179(100), 175(0.29), 149(19.9), 165(0.87)	1.04
21	13.47	13.48	Sweroside ^a,b,^*	C_17_H_23_O_11_	[M − H + HCOOH]^−^	403.12349	403.12506	403.12488	1.572	1.392	357(100), 339(1.06), 283(0.21), 267(4.96), 195(45.04), 177(0.50), 180(2.67), 151(8.32), 125(16.99)	357(100), 339(1.39), 267(5), 195(48.96), 177(1.76), 180(1.24), 151(6.9), 125(17.08), 119(0.19)	0.99
22	13.57		Loganin *	C_18_H_27_O_12_	[M − H + HCOOH]^−^	435.14970	435.15146		4.039		389(12.53), 227(100), 371(0.25), 209(0.27), 127(0.31); (Standard)		-
23	13.87	13.90	Methylcorymbiferin ^a,b^ [27]	C_18_H_17_O_9_	[M − H + CH_3_COOH]^−^	377.08671	377.08704	377.08734	0.879	1.675	362(0.1), 347(0.22), 197(100), 179(0.61), 153(17.46)	359(0.11), 347(0.6), 197(100), 179(0.41), 153(23.02)	1.92
24	13.92	13.96	1-hydroxy-2,3,4,7-tetramethoxy xanthone ^a,b^ [28]	C_18_H_17_O_9_	[M − H + HCOOH]^−^	377.08671	377.08716	377.08698	1.197	0.720	347(0.48), 331(0.03), 197(100), 179(0.03), 153(20.32), 119(0.06)	347(1.46), 331(0.06), 197(100), 179(0.45), 153(21.29)	2.07
25	14.85	14.85	Isovitexin-2′′-4′-*O*-B-d-glucosyle ^a,b^ [29]	C_34_H_41_O_22_	[M − H + HCOOH]^−^	801.20840	801.21179	801.21118	3.391	2.781	765(0.69), 755(0.2), 681(0.85), 639(100), 635(0.13), 621(0.2), 477(42.58)	765(0.17), 755(0.45), 681(1.49), 639(100), 621(3.91), 477(45)	0.82
26	15.11	15.11	Gentiotrifloroside ^a,b^ [30]	C_29_H_35_O_17_	[M − H]^−^	655.18688	655.18951	655.18927	4.020	3.654	637(0.19), 493(67.72), 475(0.02), 315(100), 195(0.14)	637(0.05), 493(65.72), 475(0.22), 315(100), 339(0.23)	0.85
27	15.38	15.38	Isoorientin ^a,b^	C_21_H_21_O_11_	[M + H]^+^	449.10784	449.10727	449.10684	0.568	0.998	431(100), 413(25.4), 395(19.26), 329(27.53), 299(2.14)	431(100), 413(21.6), 395(18.98), 329(26.97), 299(3.35)	1.03
28	16.26	16.27	Isovitexin-2′′-*O*-B-d-glucosyle ^a,b^ [29]	C_28_H_31_O_17_	[M − H + HCOOH]^−^	639.15558	639.15826	639.15808	2.684	2.504	593(0.07), 431(0.03), 477(100), 357(0.2), 311(0.1), 281(0.04)	593(0.01), 431(0.01), 477(100), 357(0.12), 387(0.04)	1.05
29	17.03	17.01	Glucosyl-1-decussatin ^a,b^ [21]	C_24_H_27_O_13_	[M − H + CH_3_COOH]^−^	523.14462	523.14722	523.14703	4.975	4.612	487(0.37), 451(1.73), 371(1.12), 359(4.1), 361(74.13), 343(1.29), 299(0.3), 241(100), 165(6.39)	505(1.55), 371(1.39), 359(0.89), 361(65.37), 343(0.96), 299(1.23), 241(100), 165(8.09)	0.13
30	17.30		Isovitexin *	C_21_H_19_O_10_	[M − H]^−^	431.09727	431.09903		4.075		413(6.31), 395(1.19), 311(100), 281(0.06), 283(0.49) (Standard)		-
31	18.06	-	2′-feruloy loganin ^a^ [31]	C_27_H_35_O_13_	[M + H]^+^	567.20722	567.20477	-	2.447	-	549(100), 471(0.03), 453(7.21), 435(44.55), 229(0.13), 211(0.04)	-	-
32	-	18.42	Gentioside ^b^ [25]	C_25_H_28_O_14_N	[M + NH_4_]^+^	570.18173	-	570.18396	-	3.909	-	553(0.82), 525(0.22), 507(0.35), 287(0.37), 283(100), 253(0.2)	-
33	18.49	18.50	1-hydroxy-2-methoxy-7-*O*-primeveroylxanthone ^a,b^ [32]	C_25_H_32_O_14_N	[M + NH_4_]^+^	570.18173	570.18060	570.18390	1.984	3.804	525(2.02), 507(2.16), 489(0.79), 283(100), 265(8.27)	569(0.4), 525(3.53), 507(0.47), 283(100), 281(1.21), 265(9.85)	4.49
34	-	18.53	Desmethylbellidifolin ^b^ [33]	C_13_H_8_O_6_K	[M + K]^+^	298.99525	-	298.99619	-	0.944	-	299(0.08), 271(0.18), 255(0.47), 155(100), 137(97.69)	-
35	-	18.61	Norswertianin ^b^ [34]	C_13_H_8_O_6_K	[M + K]^+^	298.99525		298.99518	-	0.546		299(0.13), 271(0.26), 255(0.3), 155(100), 137(90.32)	-
36	18.70	18.67	6-*C*-B-d-glucospranosyltricine (isopyrenine) ^a,b^ [29]	C_23_H_25_O_12_	[M + H]^+^	493.13405	493.13306	493.13287	2.013	2.398	475(13.75), 457(3.99), 313(2.4), 315(0.49), 195(100)	475(23.34), 457(8.67), 313(5.05), 315(0.61), 195(100)	0.83
37	18.70	18.67	Gentiakochianoside ^a,b^ [21]	C_25_H_32_O_15_N	[M + NH_4_]^+^	586.17665	586.17798	586.17841	2.277	3.070	569(6.04), 551(1.89), 541(19.3), 539(8.52), 299(100), 243(24.99)	569(4.57), 551(1.81), 541(19.98), 523(1.11), 299(100), 243(51.24)	0.27
38	18.71	18.70	Isopyrenine-7-*O*-glucosyle ^a,b^ [29]	C_29_H_33_O_17_	[M − H]^−^	653.17123	653.17395	653.17328	2.724	2.050	491(8.45), 477(13.82), 371(0.06), 357(3.86), 315(100)	635(0.02), 491(4.23), 477(11.71), 371(0.05), 357(4.73), 315(100)	0.85
39	18.74	-	Isoscoparine-7-*O*-B-d- glucosyle ^a^	C_30_H_35_O_18_	[M − H + CH_3_COOH]^−^	683.18179	683.18146	-	0.484	-	655(0.06), 647(0.04), 609(0.04), 521(100), 503(0.22), 433(0.82), 311(0.4), 297(0.04)	-	-
40	19.00	19.01	1-*O*-glucosyl corymbiferin ^a,b^ [27]	C_22_H_23_O_14_	[M − H + HCOOH]^−^	511.10823	511.10571	511.10580	4.934	4.758	467(6.46), 465(4.78), 305(1.05), 297(6.36), 153(100)	493(0.3), 475(0.5), 467(4.07), 465(9.51), 349(0.31), 305(0.47), 297(5.73), 153(100)	0.62
41	20.31	20.32	3′-acetyl swerside ^a,b^ [35]	C_18_H_25_O_11_	[M + H]^+^	401.14422	401.14362	401.14392	1.504	0.756	383(4.28), 365(0.24), 341(0.44), 197(100), 179(9.18), 151(2.36), 127(7.14)	383(7.53), 365(0.5), 341(0.87), 197(100), 179(9.83), 151(2.24), 127(8.11)	1.20
42	20.89	-	4′-*O*-B-d-glucospranosyl-2′′-*O*-[1-*O*-B-d-glucosyl-2,4,4-trihydroxy-(*E*)-cinnamoyl]-2′′-isoorientin ^a^ [36]	C_44_H_49_O_27_	[M − H + CH_3_COOH]^−^	1009.24557	1009.24139	-	4.144	-	991(0.26), 973(0.02), 949(0.01), 931(0.01), 847(3.35), 846(100), 489(0.02),459(0.01), 357(0.12)	-	-
43	-	21.09	1,3,4-trihydroxy-8-β-d-glucospranosyl-5,6,7,8-tetrahydroxanthone ^b^ [37]	C_19_H_26_O_11_N	[M + NH_4_]^+^	444.15004	-	444.15210	-	4.654	-	427(0.61), 426(0.39), 399(1.64), 381(0.3), 219(100), 237(6.86)	-
44	21.99	22.00	7-hydroxy-2-methoxy-1-*O*-primeveroyl xanthone ^a,b^ [32]	C_25_H_32_O_14_N	[M + NH_4_]^+^	570.18173	570.18390	570.18054	3.804	2.089	553(1.52), 525(100), 283(38.75), 265(18.89), 229(0.71), 243(12.78)	553(0.08), 525(100), 283(33.52), 265(21.99), 229(0.22), 243(17.04)	1.03
45	22.53	22.55	Morroniside ^a,b^ [38]	C_17_H_27_O_11_	[M + H]^+^	407.15429	407.15421	407.15466	1.125	0.314	389(100), 371(0.01), 329(0.02), 245(0.39), 227(0.05), 185(0.23), 167(0.01)	389(100), 371(0.03),329(0.01), 245(0.24), 227(0.09), 185(0.32), 167(0.01)	3.05
46	-	22.71	Isoscoparine	C_24_H_25_O_13_	[M − H + CH_3_COOH]^−^	521.12897	-	521.12724	-	1.653	-	503(0.37), 485(0.77), 477(2.69), 447(0.05), 341(0.21), 315(100, 297(4.59), 195(4.36), 163(6.76)	-
47	22.93	-	8-*O*-glucosyl bellidifolin ^a^ [27]	C_20_H_20_O_10_Na	[M + Na]^+^	443.09487	443.09613	-	2.848	-	425(16.51), 407(0.4), 389(1.14), 281(1.04), 263(0.36), 247(100)	-	-
48	23.21	-	Gentianaside ^a^ [15]	C_24_H_44_O_14_Na	[M + Na]^+^	579.26232	579.26117	-	1.997	-	561(17.21), 543(0.09), 417(4.89), 399(0.31), 383(100), 255(0.08), 237(0.44)	-	-
49	23.36	-	Scabran G4 ^a^ [19]	C_34_H_49_O_24_	[M − H]^−^	841.26083	841.26135	-	1.356	-	841(11.54), 679(0.71), 517(4.48), 489(0.08), 477(100), 337(0.13)	-	-
50	23.60	23.59	Rindoside ^a,b^ [19]	C_35_H_41_O_21_	[M − H]^−^	797.21348	797.21667	797.21637	3.996	3.620	779(0.07), 761(0.02), 755(93.15), 737(7.59), 677(0.15), 635(100), 593(50.8), 575(3.7), 515(0.6), 455(0.84), 357(0.16), 315(33.53)	779(0.27), 761(0.14), 755(97.62), 737(7.45), 677(0.28), 635(100), 593(49.99), 575(4.07), 515(0.6), 455(0.54), 357(0.03), 315(34.36)	0.92
51	23.72	23.72	Macrophylloside A ^a,b^ [24]	C_40_H_43_O_22_	[M − H]^−^	875.22405	875.22638	875.22614	2.663	3.413	857(0.06), 815(0.1), 739(100), 713(0.1), 679(1.07), 653(0.36), 577(39.22), 517(1.57), 441(0.02), 315(2.68)	815(0.77), 739(100), 713(0.01), 679(1.11), 653(0.37), 577(39.1), 517(1.78), 315(3.68)	0.77
52	23.78	23.78	Depressoside ^a,b^ [39]	C_35_H_41_O_20_	[M − H]^−^	781.21857	781.22137	781.22119	3.578	3.365	619(100), 601(0.17), 655(8.23), 493(3.59), 313(0.02)	619(100), 601(0.15), 655(7.98), 493(3.13), 313(0.1)	0.89
53	25.43	25.40	Oleanolic acid ^a,b^	C_30_H_47_O_3_	[M − H]^−^	455.35197	455.35406	455.35413	2.784	2.812	455(0.3), 437(13.83), 411(1.7), 409(0.76), 233(0.85), 221(28.05), 189(100), 75(1.12)	437(9.56), 411(1.28), 409(0.68), 233(0.57), 221(27.31), 189(100), 75(1.57)	0.57
54	-	25.48	Primeverosyl-1- decussatin ^b^ [21]	C_27_H_31_O_15_	[M − H]^−^	595.16575	-	595.16437	-	2.313	-	577(1.62), 559(0.6), 549(2.54), 495(0.43), 417(0.43), 415(29.63), 279(100)	-

Note: ^a^ identified as compound in crude *Gentiana radix*; ^b^ identified as compound in wine-processed *Gentiana radix*; * identified as compound in standards.
